# Gram-negative bacteria act as a reservoir for aminoglycoside antibiotics that interact with host factors to enhance bacterial killing in a mouse model of pneumonia

**DOI:** 10.1093/femsmc/xtac016

**Published:** 2022-05-13

**Authors:** Christiaan D M Wijers, Ly Pham, Martin V Douglass, Eric P Skaar, Lauren D Palmer, Michael J Noto

**Affiliations:** Department of Pathology, Microbiology, and Immunology, Vanderbilt University Medical Center, 1161 21st Avenue South, Nashville, TN 37232, United States; Vanderbilt Institute for Infection, Immunology, and Inflammation, Vanderbilt University Medical Center, 1161 21st Avenue South, Nashville, TN 37232, United States; Department of Pathology, Microbiology, and Immunology, Vanderbilt University Medical Center, 1161 21st Avenue South, Nashville, TN 37232, United States; Vanderbilt Institute for Infection, Immunology, and Inflammation, Vanderbilt University Medical Center, 1161 21st Avenue South, Nashville, TN 37232, United States; Department of Pathology, Microbiology, and Immunology, Vanderbilt University Medical Center, 1161 21st Avenue South, Nashville, TN 37232, United States; Vanderbilt Institute for Infection, Immunology, and Inflammation, Vanderbilt University Medical Center, 1161 21st Avenue South, Nashville, TN 37232, United States; Department of Pathology, Microbiology, and Immunology, Vanderbilt University Medical Center, 1161 21st Avenue South, Nashville, TN 37232, United States; Vanderbilt Institute for Infection, Immunology, and Inflammation, Vanderbilt University Medical Center, 1161 21st Avenue South, Nashville, TN 37232, United States; Department of Microbiology and Immunology, University of Illinois Chicago, 835 South Wolcott Avenue, Chicago, IL 60612, United States; Department of Pathology, Microbiology, and Immunology, Vanderbilt University Medical Center, 1161 21st Avenue South, Nashville, TN 37232, United States; Vanderbilt Institute for Infection, Immunology, and Inflammation, Vanderbilt University Medical Center, 1161 21st Avenue South, Nashville, TN 37232, United States; Department of Medicine, Vanderbilt University Medical Center, 1161 21st Avenue South, Nashville, TN 37232, United States

**Keywords:** aminoglycosides, bacterial pneumonia, Gram-negative, host–microbe interactions, antibiotics, pulmonary surfactant

## Abstract

*In vitro* exposure of multiple Gram-negative bacteria to an aminoglycoside (AG) antibiotic has previously been demonstrated to result in bacterial alterations that interact with host factors to suppress Gram-negative pneumonia. However, the mechanisms resulting in suppression are not known. Here, the hypothesis that Gram-negative bacteria bind and retain AGs, which are introduced into the lung and interact with host defenses to affect bacterial killing, was tested. Following *in vitro* exposure of one of several, pathogenic Gram-negative bacteria to the AG antibiotics kanamycin or gentamicin, AGs were detected in bacterial cell pellets (up to 208 μg/mL). Using inhibitors of AG binding and internalization, the bacterial outer membrane was implicated as the predominant kanamycin and gentamicin reservoir. Following intranasal administration of gentamicin-bound bacteria or gentamicin solution at the time of infection with live, AG-naïve bacteria, gentamicin was detected in the lungs of infected mice (up to 8 μg/g). Co-inoculation with gentamicin-bound bacteria resulted in killing of AG-naïve bacteria by up to 3-log_10_, mirroring the effects of intranasal gentamicin treatment. *In vitro* killing of AG-naïve bacteria mediated by kanamycin-bound bacteria required the presence of detergents or pulmonary surfactant, suggesting that increased bacterial killing inside the murine lung is facilitated by the detergent component of pulmonary surfactant. These findings demonstrate that Gram-negative bacteria bind and retain AGs that can interact with host-derived pulmonary surfactant to enhance bacterial killing in the lung. This may help explain why AGs appear to have unique efficacy in the lung and might expand their clinical utility.

## Teaser

Gram-negative bacteria act as a reservoir for the aminoglycoside class of antibiotics, and aminoglycoside-laden bacteria interact with host-derived pulmonary surfactant to affect bacterial killing inside the lung.

## Introduction

Aminoglycosides (AGs) comprise a class of antibiotics that inhibit peptide synthesis by binding to the 30S ribosomal subunit resulting in bacterial cell death (Krause *et al*. 2016). Polycationic AG antibiotics initially bind to anionic sites on bacterial cell envelopes (Taber *et al*. 1987, Rivera *et al*. 1988, Krause *et al*. 2016, John *et al*. 2017). In the case of Gram-negative bacteria, these anionic sites are comprised of the polar heads of phospholipids and lipopolysaccharide (LPS) or lipooligosaccharide (LOS) (Taber *et al*. 1987, John *et al*. 2017). The cationic antibiotic colistin interacts with Gram-negative cell envelopes in a manner similar to AGs (Monem *et al*. 2020), whereas LOS/LPS prevents vancomycin uptake by Gram-negative bacteria (Simpson *et al*. 2021). Therefore, the Gram-negative cell envelope affects bacterial susceptibility to different classes of antibiotics. Following binding to bacterial cell envelopes, AG uptake into the bacterial cytosol occurs in two energy-dependent phases: EDPI and EDPII. During EDPI, AGs cross the bacterial cytoplasmic membrane in a process that is dependent on the proton motive force (PMF) (Taber *et al*. 1987). Once inside the cytosol, AG antibiotics bind to bacterial ribosomes and induce mistranslation resulting in the formation of misfolded proteins. The insertion of misfolded proteins into the bacterial inner membrane increases membrane permeability and leads to the diffusion of more AG molecules into the bacterial cytosol, which is known as EDPII. (Davis *et al*. 1986). Collectively, these processes culminate in bacterial cell death.

Despite an overall decline in AG use—in part because of toxicity (Mingeot-Leclercq and Tulkens 1999, Dobie *et al*. 2006, Krause *et al*. 2016)—optimized dosing strategies and the emergence of MDR pathogens have ensured continued clinical utility of AGs in certain settings (Ferriols-Lisart and Alós-Almiñana 1996, Serio *et al*. 2018, Bhatt *et al*. 2019). AGs are frequently used to treat bacterial lung infections in patients with cystic fibrosis (CF) (Rogers *et al*. 2003). *Pseudomonas aeruginosa* is a common cause of CF pulmonary infections, and nebulized tobramycin results in increased pulmonary function, decreased bacterial density, and decreased risk of hospitalization (Ramsey *et al*. 1993, Ramsey *et al*. 1999, Ratjen *et al*. 2010). Treatment with inhaled tobramycin leads to improvements in pulmonary function even when *P. aeruginosa* isolates have increased minimum inhibitory concentration (MIC) values for tobramycin (≥ 8 mg/L), and may therefore be resistant to treatment (Ramsey *et al*. 1999). The use of inhaled AGs is also suggested for the treatment of ventilator-associated pneumonia (VAP) and hospital acquired pneumonia (HAP) caused by MDR Gram-negative pathogens that are susceptible to AG antibiotics (Kalil *et al*. 2016, Leone *et al*. 2018). By contrast, monotherapy with systemically administered AG antibiotics is not recommended for the treatment of HAP or VAP (Kalil *et al*. 2016). Systemically administered AGs have poor lung penetration, requiring high peak serum concentrations to achieve biologically active concentrations inside the lungs (Panidis *et al*. 2005, Boselli *et al*. 2007). This increases the risk of ototoxicity and nephrotoxicity (Mingeot-Leclercq and Tulkens 1999, Dobie *et al*. 2006). These findings, therefore, raise the possibility that AGs may be more effective in the lung.

The distal airways and alveolar airspaces are lined with pulmonary surfactant, which is predominately comprised of lipids and surfactant proteins (SPs) (Han and Mallampalli 2015). Pulmonary surfactant acts as a molecular detergent and prevents alveolar collapse by lowering the surface tension at the air liquid interface (Han and Mallampalli 2015). SPs, such as SP-B and SP-D, promote bacterial clearance through opsonization and have direct antibacterial properties through increasing bacterial membrane permeability (Wu *et al*. 2003, Nkadi *et al*. 2009, Han and Mallampalli 2015). Furthermore, surfactants promote bacterial AG uptake in a PMF-independent manner (Radlinski *et al*. 2019), suggesting that the detergent-rich environment of the distal airways and alveolar spaces may potentiate the antibacterial activities of AG antibiotics.

Previous work described that exposure of the human pathogen *Acinetobacter baumannii* to an AG antibiotic *in vitro* causes alterations to the bacterium that interact with host factors to achieve suppression of pneumonia caused by multiple Gram-negative bacterial pathogens (Hood-Pishchany *et al*. 2020). These findings led to the hypothesis that Gram-negative bacteria bind and retain AG antibiotics, which are introduced into the lung and interact with antibacterial host defenses to enhance bacterial killing. Interactions between AG-bound bacteria and host-derived factors may have implications for the treatment of bacterial lung infections with AG antibiotics. Specifically, it may help explain why AG antibiotics appear to be uniquely effective in the lung, and may therefore preserve or expand the clinical utility of AG antibiotics in the treatment of pneumonia. Therefore, the current work was undertaken to address this hypothesis.

## Materials and methods

### Ethics

All animal experiments were approved by the Vanderbilt University Medical Center (VUMC) Institutional Care and Use Committee and conform to policies and guidelines established by VUMC, the Animal Welfare Act, the National Institutes of Health, and the American Veterinary Medical Association.

### Bacterial strains and culture conditions

Bacterial strains and plasmids used in this study are listed in Table S1. Unless noted otherwise, kanamycin- and gentamicin-resistant (Km^R^, Gm^R^) bacteria were grown to exponential phase (3.5 hours) at 37°C with constant agitation in Lysogeny Broth (LB) supplemented with kanamycin (40 µg/mL) or gentamicin (50 µg/mL) as appropriate. By contrast, kanamycin- and gentamicin-susceptible (Km^S^, Gm^S^) bacteria were grown to exponential phase (3.5 hours) at 37°C in LB devoid of antibiotics, after which kanamycin or gentamicin were added to a final concentration of 40 µg/mL or 50 µg/mL, respectively, as appropriate. Cultures were then incubated at 37°C for an additional 3.5 hours. Exponential-phase bacteria were pelleted by centrifugation at 4200 × g for 6 minutes and washed twice with equal volumes of ice-cold phosphate-buffered saline (PBS) to remove unbound antibiotics. Bacteria were then resuspended and further diluted in PBS as required for each experiment. Where appropriate, bacterial cultures were chemically killed prior to washing with PBS by adding an equal volume of an ice-cold ethanol/acetone mixture (1:1) and incubating cultures on ice for 10 minutes. Ethanol causes membrane damage and denaturation of proteins, whereas acetone increases membrane fluidity (McDonnell and Russell 1999, Dyrda *et al*. 2019). Bacteria were then pelleted by centrifugation as above, resuspended in the same volume of fresh ethanol/acetone, and incubated on ice for 10 minutes. Killed bacteria were then washed with and diluted in PBS as described above. A portion of this workflow has been diagrammed in Fig. 5.

### Murine infection models

Wildtype (WT), female, eight-week-old C57BL/6J mice were purchased from Jackson Laboratories. To interrogate the effects of AG-exposed bacteria on the viability of co-infecting, AG-naïve bacteria *in vivo*, a murine model of *A. baumannii* pneumonia was utilized as previously described (Jacobs *et al*. 2010). To determine the relative contributions of AG internalization and AG outer membrane (OM) binding to killing of co-infecting, AG-naïve bacteria *in vivo*, Km^S^*Escherichia coli* DH5⍺ was incubated with kanamycin ± carbonyl cyanide m‐chlorophenylhydrazone (CCCP) or MgSO_4_, respectively, prior to infection. First, *E. coli* was grown until exponential phase as described above. Next, kanamycin ± CCCP or MgSO_4_ were added, and cultures were incubated for an additional 3.5 hours. *Escherichiacoli* viability was determined via serial dilution in PBS and plating on LB agar (LBA) plates immediately prior to and after incubation with kanamycin ± CCCP or MgSO_4_, followed by chemical killing and washing with PBS as described above. Finally, to test the hypothesis that the quantity of AG bound by Gram-negative bacteria is an important determinant of AG-naïve bacterial killing inside the murine lung, Km^R^*A. baumannii* was grown in and Km^S^ WT *A. baumannii* 17978 was exposed to media supplemented with various concentrations of kanamycin prior to chemical killing and washing as described above. *A. baumannii* ATCC 17978UN derivative, Tn5A7 (*∆lpsB*::Tn5), reliably induces enhanced killing of co-infecting bacteria in the lung after AG exposure independent of disruption of *lpsB* (Hood-Pishchany *et al*. 2020). Therefore, *A. baumannii* Tn5A7 was used as the Km^R^*A. baumannii* strain for these experiments.

Prior to infection, mice were anesthetized with 250–450 μL of a 2,2,2-tribromoethanol solution (25 mg/mL) via intraperitoneal injection. Adequate anesthesia was assessed for each animal by observing the absence of limb movement in response to applying pressure on the toe pads of both hind limbs. Mice were infected intranasally with 3×10^8^ cfu of WT *A. baumannii* ATCC 17978VU, which served as the AG-naïve inoculum for all animal infections, suspended in 30 µL of PBS. For co-inoculation experiments, unless stated otherwise, *A. baumannii* 17978/pMU368, *A. baumannii* Tn5A7, *E. coli* DH5⍺/pCR2.1, *P. aeruginosa* PAO1/pME260, and *Klebsiella pneumoniae* 43816/pCR2.1 (all Km^R^) were grown with or without kanamycin and subsequently chemically killed and washed as described above. Alternatively, mid-exponential-phase *E. coli* DH5⍺ or WT *A. baumannii* 17978 (both Km^S^) were exposed to media with or without kanamycin prior to chemical killing and washing. The strains used for each experiment are in the figure legends. For co-inoculation experiments, bacterial slurries (1×10^10^ cfu/mL) were mixed in a 1:1 ratio prior to intranasal challenge. As such, the total bacterial load in each challenge inoculum (3×10^8^ cfu) remained consistent. To determine how co-inoculation with gentamicin-exposed bacteria compares to intranasal treatment with gentamicin solution, mice received a second intranasal inoculum of gentamicin in PBS or PBS alone immediately following primary intranasal co-inoculation with live, AG-naïve, WT *A. baumannii* 17978VU (Gm^S^) and killed, Gm^R^*A. baumannii ∆hcp*::gm grown in media with or without gentamicin. Following infection, each inoculum was verified by serially diluting in PBS and plating on LBA for bacterial enumeration. At 36 hours post infection (h.p.i.), mice were euthanized through forced CO_2_ inhalation and lungs were harvested, submerged in 500 μL of sterile, ice-cold PBS, and homogenized in a benchtop bead beater using stainless steel beads. Lung homogenates were serially diluted in PBS and plated on LBA plates for bacterial enumeration. A portion of this workflow is diagrammed in Fig. 5.

### Measurement of kanamycin and gentamicin concentrations in bacterial cultures and mouse lung homogenates

To test the hypothesis that bacteria bind and retain AG antibiotics following *in vitro* exposure despite multiple washes, bacteria were grown in (Gm^R^/Km^R^) or exposed to (Gm^S^/Km^S^) media supplemented with kanamycin or gentamicin for 3.5 hours, killed, and washed. To determine the relative contributions of AG internalization and AG OM binding to gentamicin binding and retention by Gram-negative bacteria during *in vitro* exposure, mid-exponential-phase *E. coli* DH5⍺ or WT *A. baumannii* 17978 (both Gm^S^) were incubated with kanamycin ± CCCP or MgSO_4_, respectively. Bacterial viability was determined as described above immediately prior to and after incubation with gentamicin ± CCCP or MgSO_4_, which was followed by chemical killing and washing with PBS. To test the hypothesis that gentamicin can be detected in lung homogenates of mice co-inoculated with gentamicin-exposed bacteria, lung homogenates were centrifuged to remove debris and supernatants were collected. Gentamicin and kanamycin in cell pellets of killed bacteria and mouse lung homogenate supernatants were quantified using a competitive enzyme-linked immunoassay (ELISA) (Cell Biolabs, San Diego, CA) using the manufacturer's protocol. To corroborate AG quantification data obtained using ELISAs, kanamycin and gentamicin were quantified using liquid chromatography coupled with mass spectrometry (LC-MS) as follows. Samples were derivatized with benzoyl chloride and analyzed on a Thermo LTQ Orbitrap XL mass spectrometer by reverse phase on an Agilent Poroshell 120 EC-C18 2.7uM 3.0×50 mm column. The gradient started at 50% A (15 mM ammonium acetate + 0.2% acetic acid in 95% water and 5% methanol) and reached 100% B (15 mM ammonium acetate +0.2% acetic acid in 45% methanol, 45% acetonitrile, and 10% water) in 8 minutes and held for 2.5 minutes before returning to the starting conditions and re-equilibrated for 4.5 minutes.

### *In vitro* co-incubation experiments

Exponential-phase bacterial cultures grown in (Km^R^) or exposed to (Km^S^) media alone (LB) or media supplemented with kanamycin were prepared, and killed when appropriate, as described above. To determine the effects of co-incubation with killed, kanamycin-exposed bacteria (*A. baumannii* 17978/pMU368, *P. aeruginosa* PAO1/pME260; both Km^R^) on the viability of AG-naïve WT *A. baumannii* 17978 (Km^S^) *in vitro*, appropriate bacterial slurries (1×10^10^ cfu/mL) were mixed in a 1:1 ratio. Bacterial mixtures were incubated at 37°C with constant agitation, and the viability of AG-naïve WT *A. baumannii* was monitored over time through serial dilution in PBS and plating on LBA. To determine the effects of pulmonary surfactant and its components (detergent, SPs) on the viability of WT AG-naïve WT *A. baumannii* in the presence of killed, kanamycin-exposed *A. baumannii* TN5A7 (Km^R^), appropriate bacterial slurries were mixed as described above and resuspended in PBS supplemented with Triton X-100 (0.1%), deoxycholic acid (10 mg/mL), SP-B (5 μg/mL), SP-D (25 μg/mL), or SP-B (5 μg/mL) and SP-D (25 μg/mL) (Wu *et al*. 2003). Alternatively, an equal volume of porcine surfactant bronchoalveolar lavage fluid (BALF) (Curosurf, Chiesi, Boston, MA) was added to the bacterial suspensions for a final concentration of 50% pulmonary surfactant BALF. Samples were incubated at 37°C with constant agitation and the viability of AG-naive WT *A. baumannii* was monitored over time.

To test the hypothesis that detergents displace gentamicin from gentamicin-bound bacteria, mid-exponential-phase WT *A. baumannii* 17978 (Gm^S^) was exposed to and *A. baumannii ∆hcp*::gm (Gm^R^) was grown in media with gentamicin and subsequently killed and washed as described above. Bacterial slurries (1×10^10^ cfu/mL) were resuspended in an equal volume of PBS supplemented with deoxycholic acid (10 mg/mL), and incubated at 37°C with constant agitation for 6 hours. Following incubation, samples were centrifuged, supernatants were aspirated, and bacterial cell pellets were resuspended in an equal volume of PBS. A fraction of each sample was pelleted, resuspended in Tris-EDTA buffer, and lysed in Lysing Matrix B tubes using a FastPrep-24™ bead beating grinder (MP Biologicals, Irvine, CA). Subsequently, samples were pelleted to remove cellular debris and the soluble lysates were collected. Gentamicin in bacterial cell pellets and soluble lysates was quantified using LC-MS as described above.

### Quantification of AG binding and retention by LOS-insufficient *A. baumannii*

To determine the effects of *A. baumannii* LOS insufficiency on gentamicin binding and retention during *in vitro* exposure, LOS insufficiency was induced as follows. WT *A. baumannii* 17978 was grown overnight in the presence of vehicle (DMSO) or CHIR-090 (40 μg/mL). CHIR-090 is a pharmacological inhibitor of LpxC (Barb *et al*. 2007, Wei *et al*. 2017), which catalyzes the first committed step in lipid A synthesis (Anderson *et al*. 1988). Therefore, treatment of *A. baumannii* with CHIR-090 results in a relative insufficiency of LOS (Wei *et al*. 2017). Overnight cultures were diluted 1:100 in fresh LB supplemented with DMSO or CHIR090 (40 μg/mL) as appropriate, and incubated at 37°C with constant agitation until mid-exponential-phase (approximately 3.5 hours). Mid-exponential-phase cultures were incubated with gentamicin for an additional 3.5 hours, after which bacterial cultures were killed and washed as described above. Relative LOS insufficiency was confirmed using gel electrophoresis as follows. Cells were resuspended in LDS sample buffer supplemented with 1.5% 2-mercaptoethanol, boiled for 10 minutes, incubated overnight with proteinase K at 55°C, and boiled for an additional 10 minutes. Samples were run on a 10% Bis-Tris gel, followed by LPS/LOS staining using a Pro-Q™ Emerald 300 lipopolysaccharide gel stain kit (ThermoFisher, Waltham, MA) per the manufacturer's recommendations. LOS was quantified using ImageJ software. Gentamicin in LOS-insufficient and LOS-sufficient *A. baumannii* cell pellets was quantified using LC-MS as described above.

### Measurement of kanamycin and gentamicin MICs

Minimum inhibitory concentrations of kanamycin and gentamicin were determined by spreading 150 μL of a stationary-phase culture of the indicated strain on an LBA plate followed by the placement of an MIC test strip (Liofilchem s.r.l., Roseto degli Abruzzi TE, Italy) on the agar surface. Plates were then incubated at 37°C for 16 hours. Following incubation, MICs were determined by the intersection of the zone of growth inhibition with the test strip.

### Quantification and statistical analysis

Statistical analyses were performed using GraphPad Prism version 7. For animal infections, animals were randomly assigned to experimental groups using a GraphPad Prism random number calculator. Prior to animal experiments, power calculations were performed and powered for a 4-log_10_ difference in bacterial burden with an estimated standard deviation of 2-log_10_ and an ⍺ of 0.05. Mean comparisons were performed using unpaired Welch's *t*-test or one-way ANOVA adjusted for multiple comparisons as appropriate. *P* values less than 0.05 were considered statistically significant. Statistical details of experiments can be found in the figure legends.

## Results

### Gram-negative bacteria bind and retain AG antibiotics, which can interact with host factors in the lung to affect bacterial killing

**Figure 1.**
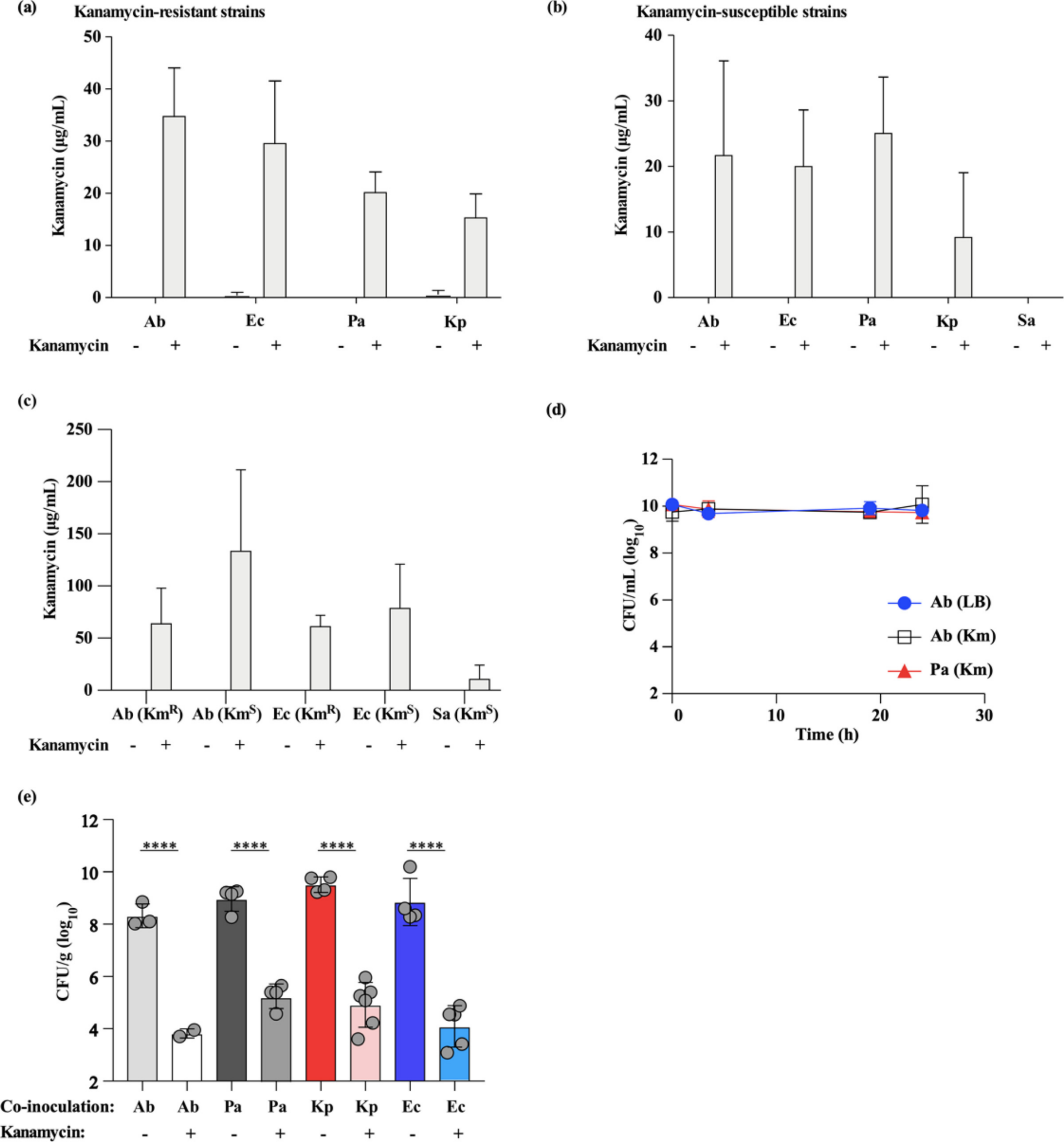
Gram-negative bacteria bind and retain AG antibiotics, which can interact with host factors in the lung to affect bacterial killing. **(a**and**b)**, The concentrations of kanamycin in cell pellets of chemically killed, kanamycin-resistant (a) *A. baumannii* 17978/pMU368 (Km MIC: 104.0 mg/L; Km^R^), *E. coli* DH5⍺/pCR2.1 (Km MIC: >256 mg/L; Km^R^), *P. aeruginosa* PAO1*/*pME260 (Km MIC: >256 mg/L; Km^R^), and *K. pneumoniae* 43816/pCR2.1 (Km MIC: 131.5 mg/L; Km^R^) or kanamycin-susceptible (b) *A. baumannii* 17978 (Km MIC: 0.9 mg/L; Km^S^), *E. coli* DH5⍺ (Km MIC: 1.25 mg/L; Km^S^), *P. aeruginosa* PAO1 (Km MIC: 10 mg/L; Km^S^), *K. pneumoniae* 43816 (Km MIC: ND; Km^S^), and *S. aureus* USA300 LAC (Km MIC: ND; Km^S^) exposed to media alone (LB) or media supplemented with kanamycin are shown as quantified by ELISA. **(c)**, The concentrations of kanamycin in cell pellets of kanamycin-resistant *A. baumannii*/pMU368 and *E. coli*/pCR2.1, and kanamycin-susceptible *A. baumannii*, *E. coli*, and *S. aureus* exposed to media alone (LB) or media supplemented with kanamycin are shown as quantified by LC-MS. **(d)**, Mid-exponential-phase WT *A. baumannii* (Km MIC: 0.9 mg/L; Km^S^) grown in media without antibiotics (LB) was co-incubated with chemically killed WT *A. baumannii* (Km^S^), *A. baumannii*/pMU368 (Km MIC: 104.0 mg/L; Km^R^), or *P. aeruginosa*/pME260 (Km MIC: >256 mg/L; Km^R^) exposed to media alone (LB) or media supplemented with kanamycin as indicated. Viability of AG-naïve, WT *A. baumannii* was monitored over time. **(e)**, Mice were infected with mid-exponential-phase WT *A. baumannii* (Km MIC: 0.9 mg/L; Km^S^) grown in media without antibiotics (LB) and co-inoculated with chemically killed *A. baumannii*/pMU368 (Km MIC: 104.0 mg/L; Km^R^), *P. aeruginosa*/pME260 (Km MIC: >256 mg/L; Km^R^)*, K. pneumoniae*/pCR2.1 (Km MIC: 131.5 mg/L; Km^R^), or *E. coli*/pCR2.1 (Km MIC: >256 mg/L; Km^R^) exposed to media alone (LB) or media supplemented with kanamycin as indicated. Bacterial burdens in the lungs of infected mice were determined at 36 h.p.i. (a-c), N = 3 biological replicates per group, per experiment. Columns depict the mean and error bars show standard deviation (a and b) or standard error (c) of the mean. (d), N = 3 biological replicates per group, per experiment. Symbols depict the mean and error bars show standard deviation of the mean. (e), Circles represent individual animals, columns depict the mean, and error bars show standard deviation of the mean. Means were compared using a one-way ANOVA adjusted for multiple comparisons. ^****^: *P* < 0.0001; ns: not significant. Ab: *Acinetobacter baumannii*; Ec: *Escherichia coli*; Pa: *Pseudomonas aeruginosa*; Kp: *Klebsiella pneumoniae*; Sa: *Staphylococcus aureus*; Km: kanamycin; ND: not determined..

To test the hypothesis that Gram-negative bacteria bind kanamycin during *in vitro* exposure and retain it despite multiple washes, *A. baumannii*, *K. pneumoniae*, *P. aeruginosa*, and *E. coli* were exposed to medium with or without kanamycin and the concentration of kanamycin in cell pellets of chemically killed bacteria was determined using a competitive ELISA. For each species, a kanamycin-resistant and a kanamycin-susceptible strain was used. The concentration of kanamycin detected in cell pellets of killed, kanamycin-resistant bacteria ranged from approximately 16 (*K. pneumoniae*) to 35 μg/mL (*A. baumannii*) (Fig. 1a). In cell pellets of killed, kanamycin-susceptible bacteria, detected kanamycin concentrations ranged from approximately 9 (*K. pneumoniae*) to 25 μg/mL (*P. aeruginosa*). No kanamycin was detected in cell pellets of the Gram-positive bacterium *Staphylococcus aureus* (Fig. 1b). Similar data were obtained using LC-MS, although low concentrations of kanamycin were detected in cell pellets of *S. aureus* using this more sensitive method (Fig. 1c). These data indicate that Gram-negative bacteria bind kanamycin during *in vitro* exposure and retain it despite multiple washes. As both kanamycin-resistant and kanamycin-susceptible bacteria are equally capable of binding and retaining kanamycin, these data indicate that the presence of an AG 3'-phosphotransferase kanamycin-resistance determinant is not required for this phenotype.

To determine if kanamycin bound to Gram-negative bacteria affects the viability of AG-naïve bacteria, unexposed *A. baumannii* was mixed with kanamycin-exposed and killed *A. baumannii* or *P. aeruginosa in vitro*. Co-incubation with kanamycin-exposed *A. baumannii* or *P. aeruginosa* did not impact the survival of AG-naïve *A. baumannii* in the mixed suspension (Fig. 1d), which is consistent with previous observations (Hood-Pishchany *et al*. 2020). To determine if kanamycin bound to Gram-negative bacteria affects the viability of AG-naïve bacteria during the course of pneumonic infection, mice were inoculated with kanamycin-exposed and killed *P. aeruginosa*, *K. pneumoniae*, or *E. coli* at the time of infection with live, AG-naïve *A. baumannii*. Inoculation with kanamycin-exposed bacteria resulted in a 4-log_10_ decrease in *A. baumannii* burdens in the lungs of infected mice, whereas inoculation with kanamycin-unexposed bacteria did not (Fig. 1e). These findings demonstrate that the reservoir of kanamycin bound to bacteria is insufficient to affect killing of AG-naïve bacteria *in vitro*, but that kanamycin bound to bacteria is sufficient to affect killing of AG-naïve bacteria in the murine lung. Therefore, these findings suggest that kanamycin bound to Gram-negative bacteria interacts with host factors in the lung to kill AG-naïve bacteria.

### Co-inoculation of mice with gentamicin-bound bacteria may be as effective as treatment of mice with inhaled gentamicin

**Figure 2.**
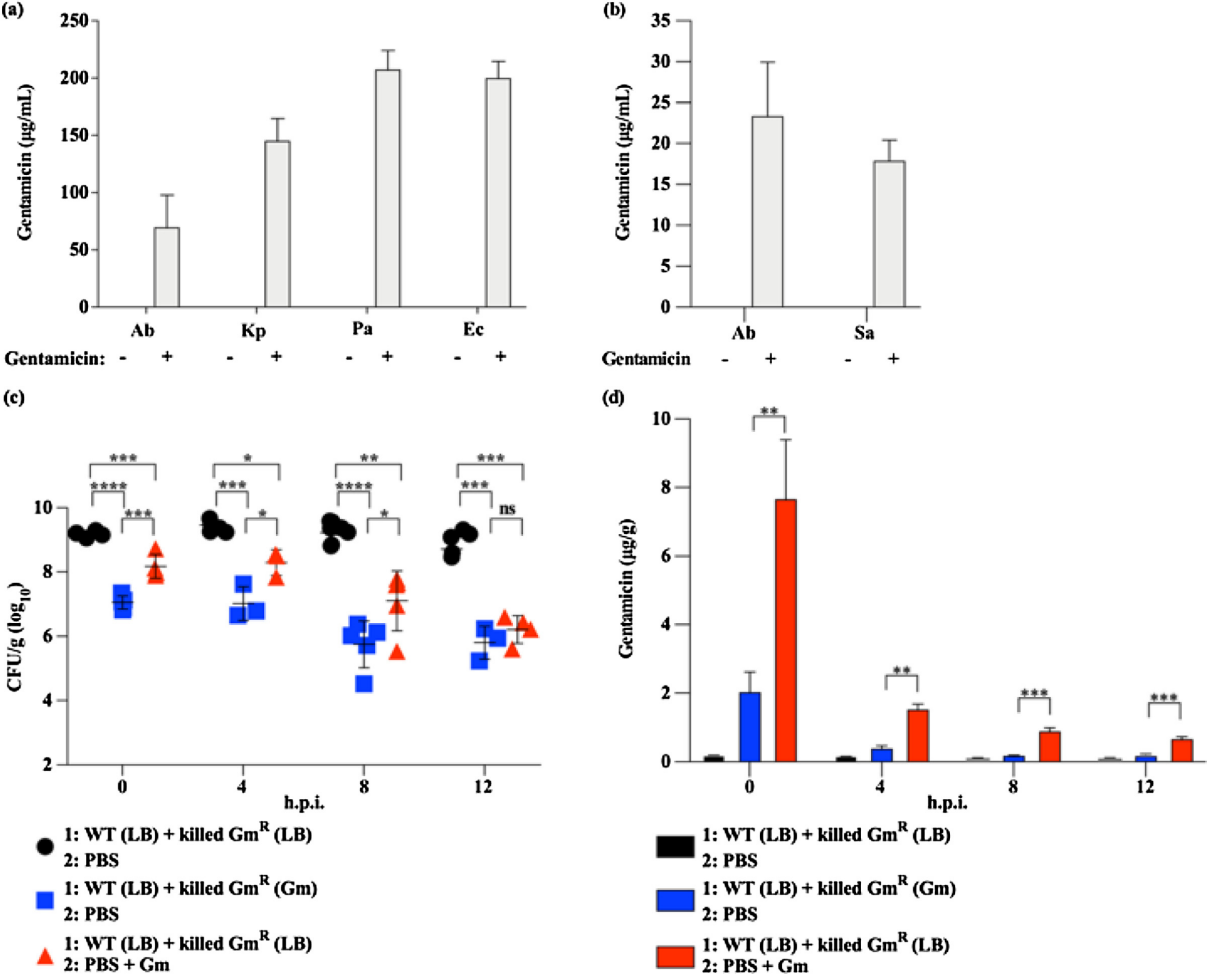
Co-inoculation of mice with AG-bound bacteria may be as effective as treatment of mice with inhaled AGs. **(a** and **b)**, The concentrations of gentamicin in cell pellets of chemically killed *A. baumannii* 17978 *∆hcp*::gm (Gm MIC: >256 mg/L; Gm^R^), *K. pneumoniae* 43816 (Gm MIC: 1.5 mg/L; Gm^S^), *P. aeruginosa* PAO1 (Gm MIC: 0.46 mg/L; Gm^S^), *E. coli* DH5⍺ (Gm MIC: 1.25 mg/L; Gm^S^), and *S. aureus* USA 300 LAC (Gm MIC: 1.5 mg/L; Gm^S^) exposed to media with or without gentamicin are shown as quantified by ELISA (a) or LC-MS (b). **(c)**, Bacterial burdens in the lungs of mice infected with mid-exponential-phase, WT *A. baumannii* 17978 (Gm MIC: 0.38 mg/L; Gm^S^) exposed to media without antibiotics (LB); co-inoculated with *A. baumannii Δhcp*::gm (Gm MIC: >256 mg/L; Gm^R^) exposed to LB ± gentamicin as indicated; and treated intranasally with PBS or PBS supplemented with gentamicin (64 μg/mL) are depicted. Bacterial burdens in the lungs of infected mice were determined at the indicated times post-infection. **(d)**, concentrations of gentamicin detected in lung homogenates of infected mice using a competitive ELISA are shown. (a and b), N = 3–4 biological replicates per group, per experiment. Columns depict the mean and error bars show standard deviation of the mean. (c), symbols represent individual animals, center bars depict the mean, and error bars show standard deviation of the mean. (d), Columns depict the mean and error bars show standard deviation of the mean. (c and d), For each time point, means were compared to all other means using a one-way ANOVA adjusted for multiple comparisons. *: *P*< 0.05; **: *P*< 0.01; ***: *P* < 0.001; ^****^: *P* < 0.0001; ns: not significant. Ab: *Acinetobacter baumannii*; Kp: *Klebsiella pneumoniae;* Pa: *Pseudomonas aeruginosa*; Ec: *Escherichia coli;* Sa: *Staphylococcus aureus*; Gm: gentamicin; h.p.i.: hours post-infection; μg/g: μg per gram of lung tissue.

To test the hypothesis that Gram-negative bacteria bind and retain AGs other than kanamycin following *in vitro* exposure, Gram-negative bacteria were exposed to gentamicin, and gentamicin concentrations in bacterial cell pellets were quantified using two distinct but complementary methods. Detected gentamicin concentrations ranged from approximately 70–208 μg/mL using a competitive ELISA (Fig. 2a). Similar data were obtained using LC-MS (Fig. 2b). These data suggest that the binding and retention of AG antibiotics by Gram-negative bacteria is generalizable across multiple AGs, including kanamycin and gentamicin. However, it was previously demonstrated that this phenotype is specific to this class of antibiotics (Hood-Pishchany *et al*. 2020).

To test the hypothesis that intranasal challenge with gentamicin-bound bacteria mimics inhalation treatment with gentamicin solution, mice were infected with live, AG-naïve *A. baumannii* and co-inoculated with killed, Gm^R^*A. baumannii* exposed to media with or without gentamicin. Immediately after infection, mice were dosed intranasally with gentamicin solution or vehicle (PBS). Mice co-inoculated with gentamicin-bound *A. baumannii* and mice treated with gentamicin solution both exhibited significant reductions in the burden of AG-naïve *A. baumannii* over time. At 0, 4, and 8 hours post-infection (h.p.i.), *A. baumannii* burdens of mice co-inoculated with gentamicin-bound *A. baumannii* were significantly lower than those of mice treated with gentamicin solution (Fig. 2c). To test the hypothesis that intranasal challenge with gentamicin-bound bacteria introduces gentamicin antibiotics into the mouse lung, the concentration of gentamicin in lung homogenates of infected mice was measured. Gentamicin was detected in lung homogenates of infected mice treated with gentamicin solution, and in lung homogenates of infected mice co-inoculated with gentamicin-bound *A. baumannii* (Fig. 2d). At 0, 4, 8, and 12 h.p.i., the gentamicin concentration was significantly greater in lung homogenates of infected mice treated with gentamicin solution, despite less bacterial killing in this group (Fig. 2c and d). These data suggest that co-inoculation with AG-bound bacteria introduces AG antibiotics into the lung and achieves bacterial killing that may be at least as potent as inhalation treatment with AG solution.

### The Gram-negative outer membrane serves as a reservoir for AG antibiotics

**Figure 3.**
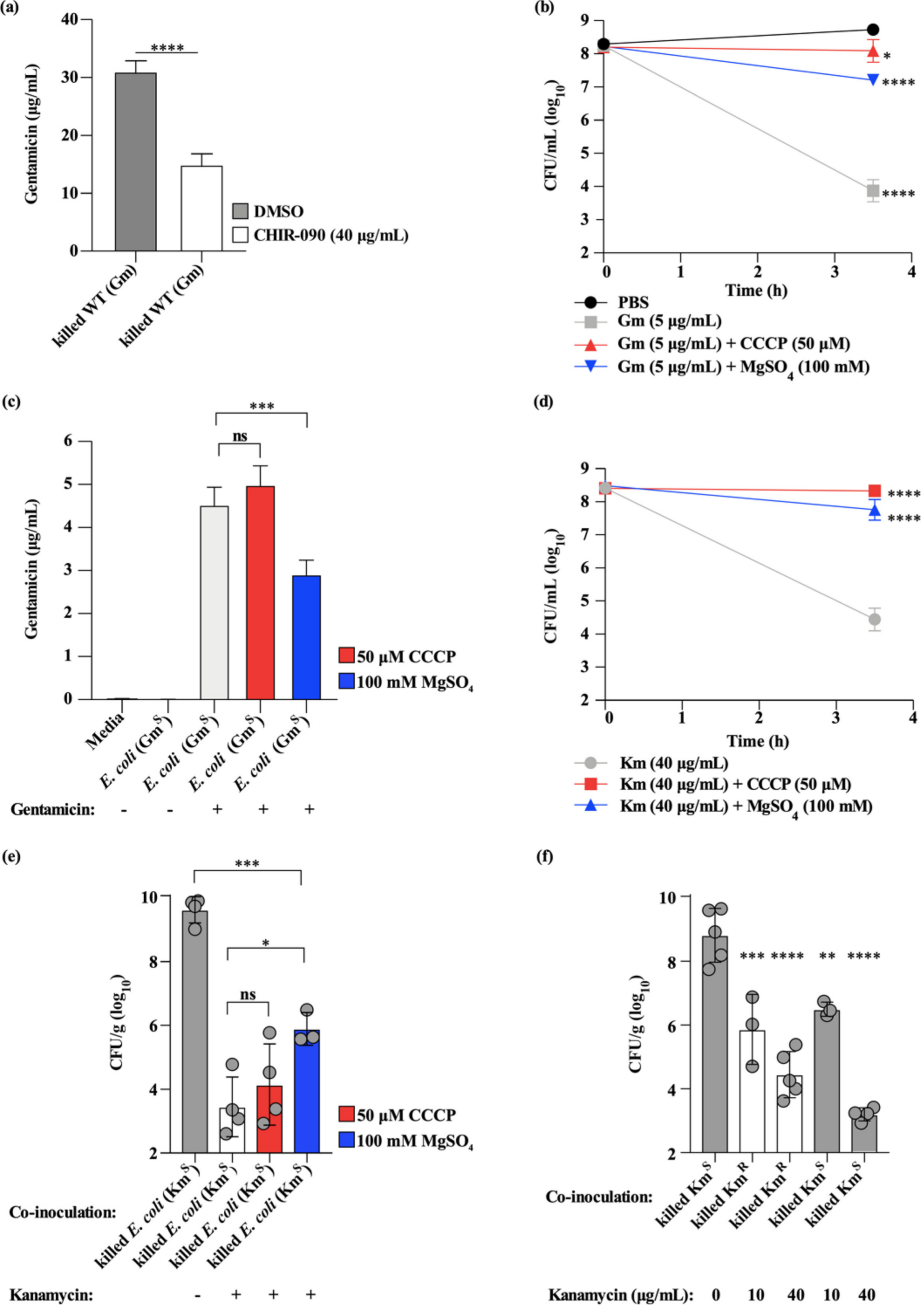
The Gram-negative outer membrane serves as a reservoir for AG antibiotics. **(a)**, The concentration of gentamicin in cell pellets of chemically killed LOS-sufficient and LOS-insufficient *A. baumannii* 17978 (Gm MIC: 0.38 mg/L; Gm^S^) exposed to media with gentamicin is shown as quantified by LC-MS **(b)**, Viability of *E. coli* DH5⍺ (Gm MIC: 1.25 mg/L; Gm^S^) exposed to PBS or gentamicin ± CCCP or MgSO_4_*in vitro* before and after exposure is depicted. **(c)**, The concentrations of gentamicin in cell pellets of chemically killed *E. coli* DH5⍺ (Gm MIC: 1.25 mg/L; Gm^S^) exposed to PBS or gentamicin ± CCCP or MgSO_4_*in vitro* are shown as quantified by ELISA. **(d)**, Viability of *E. coli* DH5⍺ (Km MIC: 1.25 mg/L; Km^S^) exposed to kanamycin ± CCCP or MgSO_4_*in vitro* before and after exposure is depicted. **(e)**, Bacterial burdens in the lungs of mice infected with mid-exponential phase WT *A. baumannii* 17978 (Km MIC: 0.9 mg/L; Km^S^) grown in media alone (LB) and co-inoculated with chemically killed *E. coli* DH5⍺ (Km MIC: 1.25 mg/L; Km^S^) exposed to kanamycin ± CCCP or MgSO_4_*in vitro* prior to infection are shown. Bacterial burdens were determined at 36 h.p.i. **(f)**, Bacterial burdens in the lungs of mice infected with mid-exponential phase, WT *A. baumannii* 17978 (Km MIC: 0.9 mg/L; Km^S^) grown in media alone (LB) and co-inoculated with chemically killed *A. baumannii* Tn5A7 (Km MIC: 128 mg/L; Km^R^) or WT *A. baumannii* 17978 (Km MIC: 0.9 mg/L; Km^S^) exposed to varying concentrations of kanamycin as indicated. Bacterial burdens were determined at 36 h.p.i. (a), N = 4–5 replicates per group, per experiment. Columns depict the mean and error bars show standard deviation of the mean. Means were compared using a Welch's *t*-test. (b and d), N = 4 (b) or N = 5 replicates (d) per group, per experiment. Symbols depict the mean and error bars show standard deviation of the mean. Means were compared to the mean bacterial viability of the untreated group (PBS) (b) or to the group treated with kanamycin alone (Km) (d) using a one-way ANOVA adjusted for multiple comparisons. (c), N = 3–4 biological replicates per group, per experiment. Columns depict the mean and error bars show standard deviation of the mean. Means were compared to all other means using a one-way ANOVA adjusted for multiple comparisons. (e and f), Circles represent individual animals, columns depict the mean, and error bars show standard deviation of the mean. Means were compared to all other means (e) or to the mean of the first column (f) using a one-way ANOVA adjusted for multiple comparisons. *: *P*< 0.05; **: *P*< 0.01; ***: *P* < 0.001; ^****^: *P* < 0.0001; ns: not significant. Km: kanamycin; Gm: gentamicin.

Gram-negative bacteria bind and retain AG antibiotics during *in vitro* exposure, which affect killing of co-infecting bacteria inside the murine lung potentially with similar efficacy to mice treated with AG inhalation (Fig. 2). Polycationic AGs bind anionic residues on the polar heads of phospholipids, LPS, and LOS on the Gram-negative OM (Taber *et al*. 1987, Rivera *et al*. 1988, Krause *et al*. 2016, John *et al*. 2017). To test the hypothesis that LOS-insufficiency decreases gentamicin binding by *A. baumannii* following *in vitro* exposure, *A. baumannii* was treated with the LpxC inhibitor CHIR-090 to induce LOS-insufficiency (Barb *et al*. 2007, Wei *et al*. 2017). Treatment with 40 μg/mL CHIR-090 resulted in a statistically significant, approximately 50%-reduction in LOS abundance as evidenced by gel electrophoresis and subsequent LPS/LOS staining (Fig. S1). Compared to vehicle-treated *A. baumannii*, the concentration of gentamicin in cell pellets of LOS-insufficient, CHIR-090-treated *A. baumannii* was significantly reduced by approximately 50%, mirroring the reduction in LOS abundance (Fig. 3a). AG binding to the Gram-negative OM can also be reduced through the addition of Mg^2+^ (Hancock 1981, Hancock *et al*. 1981), and AG internalization into the bacterial cytosol can be inhibited by dissipating the PMF with the uncoupler CCCP (Hancock 1981, Davis 1987, Fraimow *et al*. 1991, Krause *et al*. 2016, Radlinski *et al*. 2019). To test the hypothesis that the Gram-negative OM acts as the predominant AG binding and retention reservoir, Gm^S^*E. coli* or *A. baumannii* was incubated with gentamicin and treated with CCCP or MgSO_4_. The inhibition of gentamicin internalization or binding to the OM would be expected to reduce bacterial killing by gentamicin. Congruently, addition of either CCCP or MgSO_4_ significantly reduced killing of *E. coli* and *A. baumannii* by gentamicin (Fig. 3b; Fig. S2A). Relative to incubation with gentamicin alone, the addition of CCCP did not significantly alter the concentration of gentamicin detected in *E. coli* cell pellets, whereas addition of MgSO_4_ decreased the detected concentration of gentamicin by approximately one third (Fig. 3c). Collectively, these data implicate the Gram-negative OM, but not the bacterial cytosol, as the predominant bacterial AG reservoir during *in vitro* exposure.

As MgSO_4_ decreases AG binding by Gram-negative bacteria, it was hypothesized that treatment with MgSO_4_ would reduce the amount of AG introduced into the lung through the inoculation of AG-bound bacteria. To test this, Km^S^*E. coli* was incubated with kanamycin ± CCCP or MgSO_4_. Kanamycin-mediated killing *in vitro* was assessed, and bacteria were chemically killed and inoculated into the lungs of mice at the time of infection with live, AG-naïve *A. baumannii*. Consistent with data described above, treatment with either CCCP or MgSO_4_ significantly reduced *in vitro* killing of *E. coli* by kanamycin (Fig. 3d). Further, mice co-inoculated with *E. coli* incubated with kanamycin and MgSO_4_ had an approximate 3-log_10_ increase in bacteria recovered from the lung in comparison to mice co-inoculated with *E. coli* incubated with kanamycin alone. Mice co-inoculated with *E. coli* incubated with kanamycin and CCCP had bacterial lung burdens similar to those of mice co-inoculated with *E. coli* incubated with kanamycin alone (Fig. 3e). These data suggest that kanamycin binding to the OM, but not internalization to the cytosol, is required to induce kanamycin-mediated killing of AG-naïve, co-infecting bacteria in the mouse lung.

The ability of MgSO_4_ treatment to inhibit bacterial killing *in vivo* raised the hypothesis that the quantity of AG bound by Gram-negative bacteria is an important determinant of AG-naïve bacterial killing inside the murine lung. To test this, Km^R^ and Km^S^*A. baumannii* were exposed to 0, 10, or 40 μg/mL of kanamycin, killed, and inoculated into the mouse lung at the time of challenge with live, AG-naive *A. baumannii*. Co-inoculation with kanamycin-bound *A. baumannii* enhanced bacterial killing of co-infecting *A. baumannii* in the lung in a dose-dependent manner. Further, Km^R^ and Km^S^*A. baumannii* were equally effective at increasing kanamycin-mediated killing of AG-naïve *A. baumannii* (Fig. 3f). These findings suggest that the quantity of AG present in the media during *in vitro* exposure determines the degree of AG-naïve bacterial killing in the mouse lung. Additionally, these data indicate that kanamycin modification in the cytosol by the AG 3'-phosphotransferase kanamycin resistance determinant does not impair bacterial killing mediated by the OM AG reservoir.

### AG-bound bacteria interact with pulmonary surfactant to affect AG-mediated killing of co-infecting bacteria

**Figure 4.**
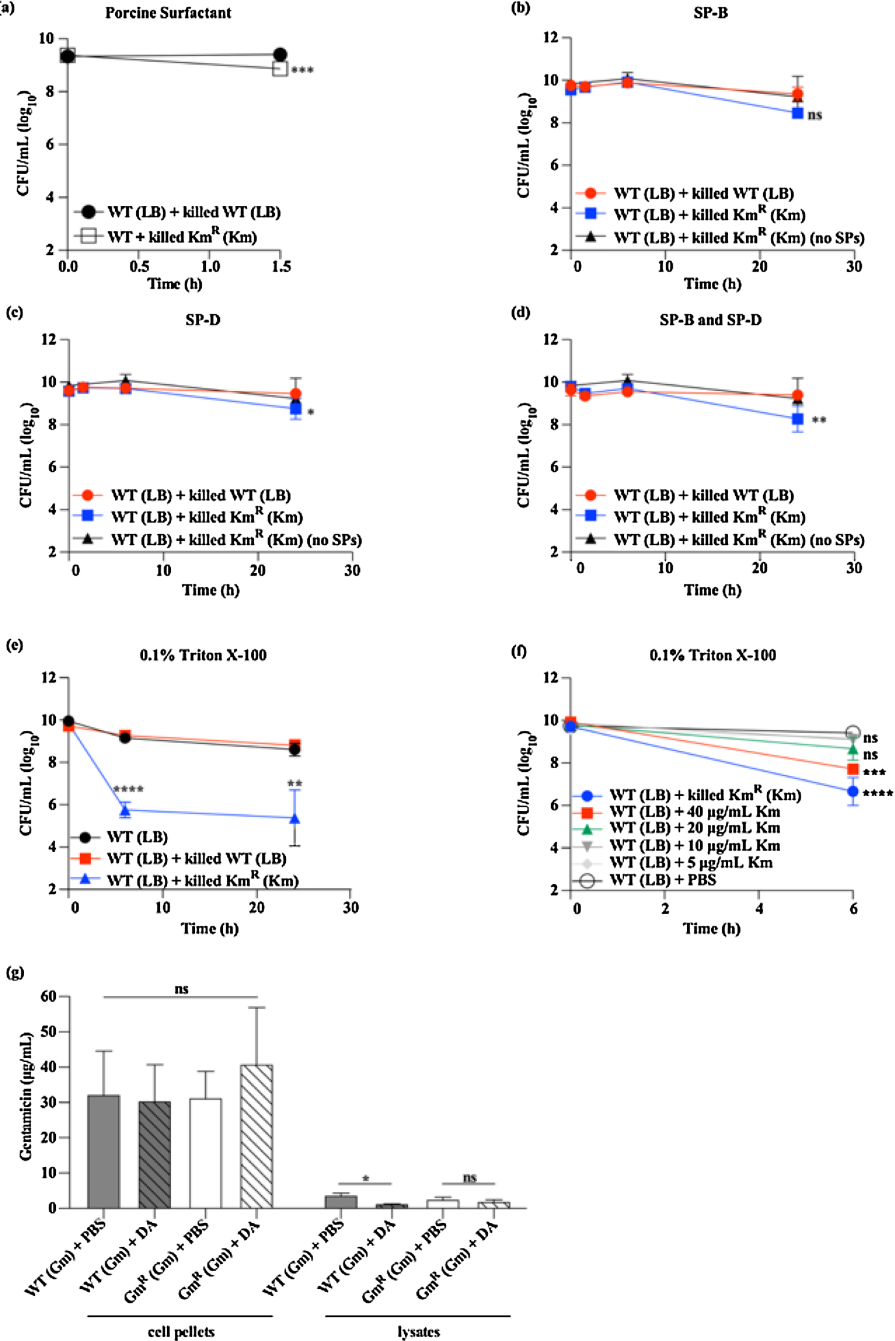
AG-bound bacteria interact with pulmonary surfactant to affect AG-mediated killing of co-infecting bacteria in the mouse lung. **(a)**, Viability of WT *A. baumannii* 17978 (Km MIC: 0.9 mg/L; Km^S^) exposed to media alone (LB) co-incubated with killed, unexposed WT *A. baumannii* 17978 (Km MIC: 0.9 mg/L; Km^S^) or killed, kanamycin-bound *A. baumannii* Tn5A7 (Km MIC: 128 mg/L; Km^R^) in the presence of 50% porcine surfactant BALF is depicted. Bacterial viability was determined immediately prior to and after incubation in porcine surfactant BALF. **(b–d)**, Viability of WT *A. baumannii* 17978 (Km MIC: 0.9 mg/L; Km^S^) exposed to media alone (LB) co-incubated with killed, WT *A*. baumannii 17978 (Km MIC: 0.9 mg/L; Km^S^) grown in media alone (LB) or killed, kanamycin-bound *A. baumannii* Tn5A7 (Km MIC: 128 mg/L; Km^R^) in the presence of 5 μg/mL SP-B (b), 25 μg/mL SP-D (c), 5 μg/mL SP-B and 25 μg/mL SP-D (d), or PBS (no SPs) is depicted. Bacterial viability was measured over time. **(e**and **f)**, Viability of WT *A. baumannii* 17978 (Km MIC: 0.9 mg/L; Km^S^), grown in media alone (LB) co-incubated with or without killed *A. baumannii* or varying concentrations of kanamycin as indicated is depicted. Where indicated, WT *A. baumannii* was co-incubated with killed, WT *A. baumannii* 17978 (Km MIC: 0.9 mg/L; Km^S^) grown in media alone (LB) or killed, kanamycin-bound *A. baumannii* Tn5A7 (Km MIC: 128 mg/L; Km^R^). Bacterial suspensions were pelleted and resuspended in PBS supplemented with 0.1% Triton X-100 and bacterial viability was monitored over time. **(g)**, The concentration of gentamicin in cell pellets and soluble lysates of killed, gentamicin-exposed WT *A. baumannii* 17978 (Gm MIC: 0.38 mg/L; Gm^S^) and *A. baumannii* 17978 *∆hcp*::gm (Gm MIC: >256 mg/L; Gm^R^) incubated with PBS alone or PBS supplemented with deoxycholic acid (10 mg/mL) as measured by LC-MS is shown. (a-g), N = 3–4 biological replicates per group, per experiment. Graphs depict average (a-d) or representative (e-g) data from at least two independent experiments. Symbols (a-f) or columns (g) depict the mean, and error bars show standard deviation of the mean. Means were compared using a Welch's *t*-test (a) or a one-way ANOVA adjusted for multiple comparisons, for the 1.5h time point (a), for the 24h time point (b-d), or for each time point (e and f). (g), means were compared using a one-way ANOVA adjusted for multiple comparisons. *: *P*< 0.05; **: *P*< 0.01; ***: *P* < 0.001; ^****^: *P* < *P* < 0.0001; ns: not significant. Km: kanamycin; Gm: gentamicin; DA: deoxycholic acid.

AG molecules are introduced by AG-bound bacteria to affect killing of co-infecting bacteria inside the mouse lung potentially with similar efficacy to inhalation treatment with AG solution (Fig. 2). However, AG-bound bacteria do not alter the viability of AG-naïve bacteria *in vitro* or in a mouse model of systemic infection (Fig. 1d and Hood-Pishchany *et al*. 2020). These findings suggest that AG-bound bacteria interact with host factors inside the mouse lung to affect bacterial killing. Pulmonary surfactant is abundant in the fluid lining the distal airways and alveolar spaces, and is encountered by bacteria upon pneumonic infection in mice (Wright *et al*. 2000, Palmer *et al*. 2020). To test the hypothesis that pulmonary surfactant combined with AG-bound bacteria affects bacterial killing, live, AG-naïve *A. baumannii* was incubated with killed, kanamycin-bound *A. baumannii* and porcine surfactant BALF, and bacterial survival was assessed. Relative to incubation with *A. baumannii* exposed to media alone (LB), incubation with kanamycin-bound *A. baumannii* resulted in a significant, approximately 50%-decrease in the number of viable AG-naïve *A. baumannii* in the presence of porcine surfactant BALF (Fig. 4a). This suggests that AG-bound bacteria interact with pulmonary surfactant to affect killing of co-infecting bacteria inside the mouse lung.

To identify the component(s) of pulmonary surfactant that interact with AG-bound bacteria to affect bacterial killing, the antibacterial effects of individual components of pulmonary surfactant combined with AG-bound bacteria were determined. Pulmonary surfactant contains several proteins with antibacterial properties, such as SP-B and SP-D (Wu *et al*. 2003, Nkadi *et al*. 2009, Han and Mallampalli 2015). In the presence of 5 μg/mL SP-B and/or 25 μg/mL SP-D (Wu *et al*. 2003), co-incubation with killed, kanamycin-bound *A. baumannii* resulted in a small decrease in viable, AG-naïve *A. baumannii* after 24 hours (Fig. 4b–d). Pulmonary surfactant is composed of 90% lipids and acts as a molecular detergent (Han and Mallampalli 2015). To test the hypothesis that detergent components of pulmonary surfactant combine with AG-bound bacteria to potentiate bacterial killing, live, AG-naïve *A. baumannii* was incubated with killed, kanamycin-bound *A. baumannii* and the nonionic detergent Triton X-100. Relative to co-incubation with killed, unexposed *A. baumannii*, co-incubation with kanamycin-bound *A. baumannii* significantly decreased the survival of AG-naïve *A. baumannii* over time (Fig. 4e). Similar results were obtained with deoxycholic acid, an antimicrobial, detergent-like bile acid (Fig. S2B) (Sistrunk *et al*. 2016). When combined with Triton X-100, AG-naïve *A. baumannii* killing increased with increasing concentrations of kanamycin, and co-incubation with kanamycin-bound *A. baumannii* was more potent than the highest concentration of kanamycin tested (Fig. 4f and S2C). These findings demonstrate that bacterial killing mediated by AG-bound bacteria is facilitated predominately by detergents—and to a lesser extent by proteins—of host-derived pulmonary surfactant.

To test the hypothesis that detergents facilitate AG-mediated killing of AG-naïve bacteria by displacing AGs from the cell envelope of AG-bound bacteria, gentamicin-bound *A. baumannii* was incubated with or without deoxycholic acid. Following incubation, the concentration of gentamicin in bacterial cell pellets and soluble lysates was quantified using LC-MS. In comparison to incubation in vehicle alone (PBS), incubation in deoxycholic acid did not significantly alter the concentration of gentamicin in cell pellets of gentamicin-bound bacteria for both Gm^S^ and Gm^R^*A. baumannii* (Fig. 4g). Further, the concentrations of gentamicin detected in bacterial cell lysates were approximately 10% of those detected in bacterial cell pellets (Fig. 4g). Treatment with detergent resulted in a small decrease in the amount of gentamicin recovered from bacterial cell lysates for both Gm^S^ and Gm^R^*A. baumannii*. In the case of Gm^S^*A. baumannii*, this decrease was statistically significant (Fig. 4g). These findings do not support the conclusion that detergents liberate AG molecules from AG-bound bacteria, but may suggest that detergents facilitate AG-mediated killing of AG-naïve bacteria by some other mechanism. These data provide additional evidence that the Gram-negative bacterial cytosol—which comprise the lysates used in this experiment—is a minor contributor to the Gram-negative AG reservoir. Collectively, these data suggest that interactions between AG-bound bacteria and pulmonary surfactant affect bacterial killing in the murine lung.

## Discussion

**Figure 5.**
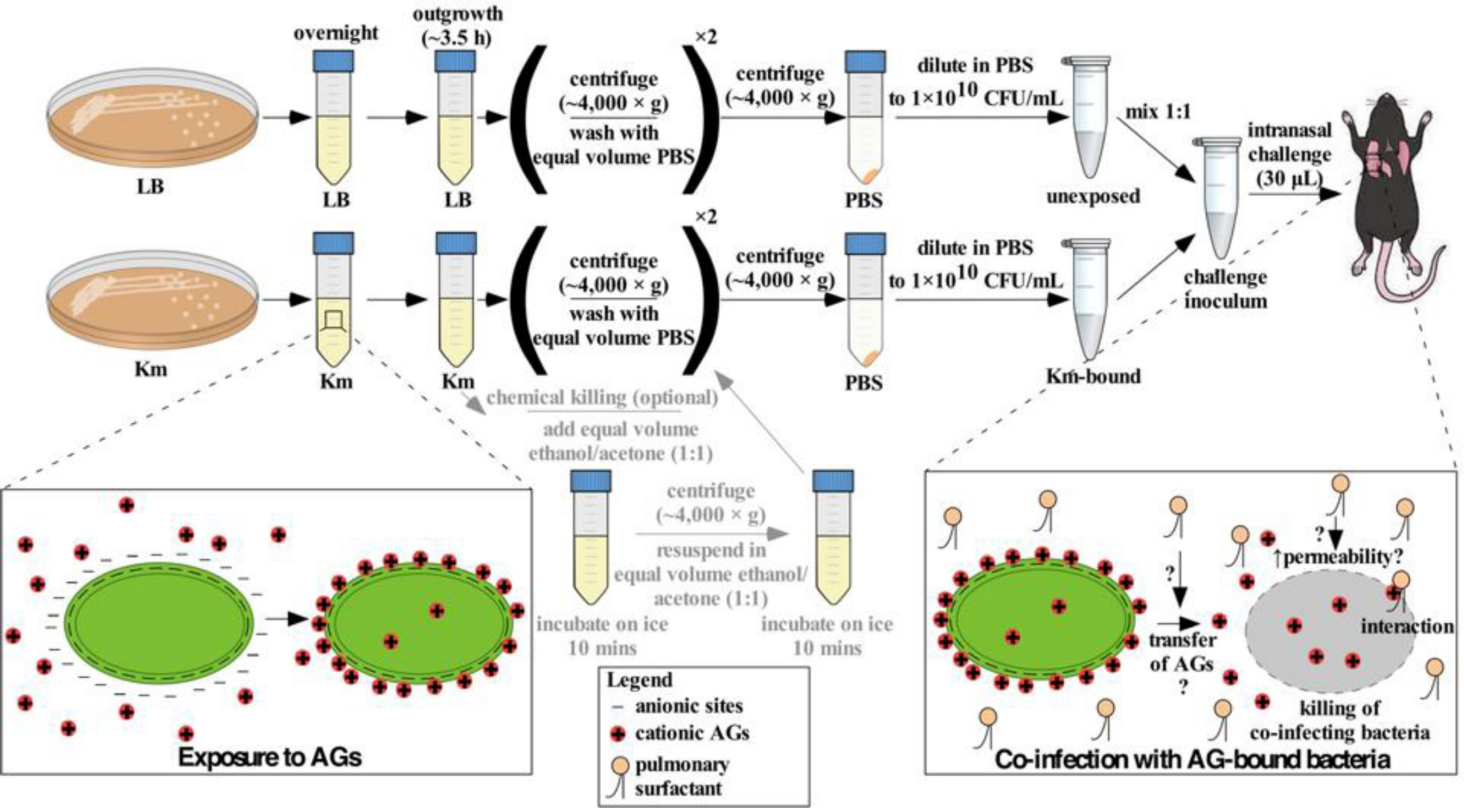
Working model of killing of co-infecting bacteria inside the murine lung mediated by AG-bound bacteria. Prior to intranasal challenge of mice, bacteria are grown in media alone (LB) or media supplemented with kanamycin (Km), washed, and diluted in PBS to 1×10^10^ cfu/mL. For co-infections and co-inoculations, bacterial suspensions (at 1×10^10^ cfu/mL) are mixed in a 1:1 ratio. During AG exposure, Gram-negative bacteria bind bioactive AG molecules to their OM which are retained despite multiple washes. Inside the mouse lung, AG-bound bacteria interact with pulmonary surfactant to affect killing of susceptible, co-infecting bacteria.

The findings presented herein support a model by which the Gram-negative OM binds and retains AG molecules, that AGs are introduced into the lung by AG-bound bacteria, and that these AGs affect killing of AG-naïve bacteria (Fig. 5). AG-bound bacteria retain kanamycin and gentamicin on the order of tens of μgs per mL of bacterial cell suspension. Therefore, AG-bound bacteria may act as an efficient drug delivery system, creating high local concentrations of AGs inside the lung. It is conceivable that local drug concentrations inside the lungs of mice co-inoculated with AG-bound bacteria are sufficiently high to cover the kanamycin or gentamicin MIC of AG-naïve, co-infecting *A. baumannii* used in the animal infections in this study (0.9 mg/L and 0.38 mg/L, respectively; Table S1). However, *in vitro* susceptibility of AG-naïve, co-infecting bacteria to AGs is not required for enhanced bacterial killing inside the murine lung mediated by AG-bound bacteria. Previous work indicates that co-inoculation of mice with kanamycin-bound bacteria at the time of infection with live, kanamycin-naïve *A. baumannii* AB5075 (kanamycin MIC: >256 mg/L; Km^R^) significantly increased *A. baumannii* AB5075 killing compared to co-inoculation with bacteria unexposed to any antibiotics (Hood-Pishchany *et al*. 2020). Therefore, the presence of a bacterial AG reservoir large enough to overcome the MIC of the co-infecting strain alone may not explain the phenotype observed. The finding that AGs are bound and retained by exposed bacteria at high levels despite multiple washes was not expected. However, a labeled derivative of the AG neomycin binds OMs in a saturable fashion, and these interactions are strong enough to withstand multiple washes (Sabeti Azad *et al*., 2020). Therefore, these findings suggest that the electrostatic interactions between cationic AGs and negatively charged bacterial OMs are strong enough to withstand multiple washes and that the OM may act as a reservoir for cationic small molecules such as AGs.

The present study expands on the observation that AGs continue to kill bacteria after the antibiotic itself is removed—the so-called post-antibiotic effect (Isaksson *et al*. 1988). AGs interact with bacteria by binding to anionic sites on Gram-negative cell envelopes such as the polar heads of phospholipids and LPS (or LOS) (Taber *et al*. 1987, Rivera *et al*. 1988, Krause *et al*. 2016, John *et al*. 2017). These anionic sites have been implicated as the binding site of AG molecules responsible for the post-antibiotic effect (Jackson *et al*. 1990). Here, several lines of evidence that implicate the OM as the predominant Gram-negative reservoir for AG molecules are presented. LOS-insufficient *A. baumannii* retained significantly less gentamicin following *in vitro* exposure compared to LOS-sufficient *A. baumannii*. Further, the divalent cation Mg^2+^ stabilizes Gram-negative OMs and prevents AG binding (Ramirez-Ronda *et al*. 1975, Hancock 1981, Hancock *et al*. 1981, Taber *et al*. 1987). Addition of Mg^2+^ during AG exposure decreases the concentration of AG detected in bacterial cell pellets and inhibits the killing of co-infecting bacteria upon subsequent pneumonic infection of mice. However, addition of the uncoupler agent CCCP, which dissipates the PMF and prevents AG entry into the bacterial cytosol (Hancock 1981, Davis 1987, Fraimow *et al*. 1991), does not. Further, both AG-resistant and AG-susceptible bacteria bind and retain AGs following exposure (Figs 1a and b and 2a), and are equally capable of enhancing bacterial killing inside the mouse lung after AG-exposure (Fig. 3e and f). In the AG-resistant strains used in the present study, resistance is imparted by AG modifying enzymes (Table S1). AGs modified by bacterial enzymes have decreased binding affinity for bacterial ribosomes (Llano-Sotelo *et al*. 2002), making the cytosol an unlikely AG reservoir. Finally, the concentration of gentamicin detected in bacterial lysates devoid of cellular debris was a fraction of the gentamicin concentration detected in bacterial cell pellets. Although these findings are most consistent with the OM being the major reservoir for AG antibiotics, bacterial uptake of AGs can occur in the absence of the proton motive force (Bruni and Kralj 2020). Therefore, some contribution of the bacterial cytosol to AG binding and retention cannot be completely excluded.

In contrast to Gram-negative bacteria, AG binding and retention by the Gram-positive pathogen *S. aureus* differed based on the specific AG antibiotic tested, as *S. aureus* bound gentamicin to a greater extent than kanamycin following *in vitro* exposure. Detection of residual kanamycin in *S. aureus* cell pellets could be due to incomplete washing. This finding suggests that AG binding by *S. aureus* may be restricted to fewer types of AG antibiotics, or to gentamicin specifically. Since all AGs are cationic, molecular properties of gentamicin other than its positive electrostatic charge may promote binding and retention by *S. aureus*. The subcellular location of the Gram-positive gentamicin reservoir remains to be identified. *S. aureus* cells have a modest net negative charge, which is increased in mutants with altered teichoic acid structure (Peschel *et al*. 1999). Therefore, to what extent the Gram-positive cell envelope contributes to the *S. aureus* gentamicin reservoir and to what extent it is capable of binding AGs other than gentamicin may differ based on teichoic acid structure.

The present study provides evidence that killing of co-infecting bacteria inside the mouse lung mediated by AG-bound bacteria may be facilitated by pulmonary surfactant, in particular its detergent components. The difference in AG-naïve bacterial survival between co-incubation in porcine surfactant BALF and co-incubation in detergents (Triton X-100, deoxycholic acid) may be due to the fact that the porcine surfactant used in this study is BALF obtained by porcine lung lavage. Therefore, the porcine surfactant is diluted and the resulting detergent suspension is likely far less concentrated than the Triton X-100 or deoxycholic acid solutions used in this study, and less concentrated than what is encountered inside the murine lung. Several detergents facilitated AG-mediated killing of AG-naïve bacteria *in vitro*, although the detergent deoxycholic acid did not liberate gentamicin from gentamicin-bound bacteria (Fig. 4g). This finding does not support the conclusion that the detergent components of pulmonary surfactant exert their effects by displacing AGs from AG-bound bacteria. Instead, pulmonary surfactant may act on AG-naïve, co-infecting bacteria by permeabilizing their cell envelopes, thereby promoting entry of AGs introduced into the mouse lung by AG-bound bacteria. This notion is consistent with previous reports demonstrating that molecular detergents increase bacterial susceptibility to AG antibiotics by increasing bacterial membrane permeability (Radlinski *et al*. 2019). The minor effect of SPs on AG-mediated killing of AG-naïve bacteria *in vitro* may be explained by a similar mechanism, as SP-A and SP-D increase bacterial membrane permeability as well (Wu *et al*. 2003). Pulmonary surfactant may facilitate the transfer of AG molecules from AG-bound bacteria to AG-naïve, co-infecting bacteria through some other mechanism that is yet to be identified. Previous work by our group has demonstrated that the detergent sodium dodecyl sulfate (SDS) does not facilitate AG-mediated killing of AG-naïve *A. baumannii in vitro* (Hood-Pishchany *et al*. 2020). In contrast to the non-ionic detergent Triton X-100, SDS is anionic. Due to their net negative surface charge, interactions between Gram-negative bacteria and anionic detergents are likely reduced relative to non-ionic detergents, thereby preventing AG-mediated bacterial killing. A more thorough understanding of the molecular interactions between pulmonary surfactant and AG-naïve or AG-bound bacteria may help explain why AG-mediated killing of co-infecting bacteria inside the mouse lung may be as or more effective when mice are co-inoculated with AG-bound bacteria as opposed to AGs in solution, despite AG concentrations being higher in lung homogenates of the latter group (Fig. 2). As this finding suggests that the greatest efficiency of bacterial killing inside the murine lung might be achieved when AGs are bound to bacteria, a possible contribution of unidentified bacterial factors cannot be excluded. Similarly, a potential role for additional host-derived factors cannot be excluded.

This work may help explain why AGs are more often used to treat bacterial lung infections relative to bacterial infections of other organ systems. Inhaled AGs (with or without the addition of systemic antibiotics) are suggested for the treatment of VAP or HAP caused by multi-drug resistant (MDR) Gram-negative pathogens that are susceptible to AG antibiotics (Kalil *et al*. 2016, Leone *et al*. 2018). Systemically administered AGs have poor lung penetration (Panidis *et al*. 2005, Boselli *et al*. 2007), and inhalation treatment with nebulized AGs likely achieves higher local drug concentrations inside the lung more effectively. A similar mechanism may be implicated in mice co-inoculated with AG-bound bacteria. By contrast, in patients with urinary tract infections, AGs are equally as effective as beta-lactams or quinolones in achieving clinical improvement, but are associated with higher rates of bacteriological failure at the end of treatment (Vidal *et al*. 2007). This is in spite of the fact that parenterally administered AGs are secreted into the urine at high concentrations (Naber *et al*. 1973, Wood and Farrell 1976). In patients with bacteremia, use of an AG instead of or in addition to a beta-lactam does not improve cure rates or reduce the risk of mortality, but does increase the risk of adverse events such as nephrotoxicity (Gudiol *et al*. 1986, Paul *et al*. 2004, Vidal *et al*. 2007, Bliziotis *et al*. 2011). This may be due to ineffective penetration of the nidus of infection, located outside the vasculature, by systemically administered AGs. These data are consistent with previous work demonstrating that co-inoculation with AG-bound bacteria does not increase killing of co-infecting bacteria in a mouse model of systemic infection (Hood-Pishchany *et al*. 2020). In patients with CF, treatment with inhaled AGs for bacterial lung infections has clinical benefits even if infecting isolates exhibit elevated MICs suggestive of *in vitro* resistance (≥ 8 mg/L) (Ramsey *et al*. 1999). Patients with CF are often colonized by a multitude of bacterial species with varying antibiotic resistance profiles, resulting in polymicrobial infections of the respiratory system (Foweraker *et al*. 2005, Zhao *et al*. 2012, Clark *et al*. 2015, Flynn *et al*. 2020, Khanolkar *et al*. 2020). The present study raises the hypothesis that AG-resistant strains within the CF lung may bind and retain bioactive AG molecules during treatment with inhaled AGs, which could then kill susceptible, co-infecting organisms. Alternatively, the combination of high local drug concentrations and pulmonary surfactant may sensitize infecting organisms that demonstrate *in vitro* resistance. This is consistent with the prior observation that co-inoculation with kanamycin-bound bacteria may increase bacterial killing even if the co-infecting strain has an elevated kanamycin MIC (> 40 mg/L) (Hood-Pishchany *et al*. 2020). Thus, the present study may preserve the clinical utility of AG antibiotics as they are currently used in the treatment of HAP and VAP, as well as potentially expand their utility to treatment of pneumonia caused by bacteria with *in vitro* resistance to AGs.

Limitations of the present study include a lack of definitive evidence confirming the role for pulmonary surfactant interactions with AGs in facilitating bacterial killing in the lungs of mice. However, as mice deficient in pulmonary surfactant phospholipid synthesis exhibit respiratory distress and perinatal mortality, the impact of the loss of pulmonary surfactant during bacterial pneumonia cannot be ascertained using this model system (Bridges *et al*. 2010). Similarly, AG binding and retention by bacterial OMs was not visualized directly. However, a recent study demonstrated that a fluorescent derivative of neomycin interacts with bacterial OMs (Sabeti Azad *et al*., 2020). Further, gentamicin in the lungs of mice co-inoculated with gentamicin-bound bacteria or gentamicin solution was quantified using lung homogenates. Therefore, the exact location of gentamicin within the lungs of mice co-inoculated with gentamicin-bound bacteria or gentamicin solution remains to be investigated. To what extent gentamicin is unbound and freely available within the lungs of these mice remains to be definitively determined as well. As AGs are predominately distributed extracellularly, lung homogenate gentamicin concentrations may underestimate the concentration of freely available gentamicin present in the alveolar air spaces and distal airways (Mouton *et al*. 2008). Therefore, it remains to be fully investigated whether AG-bound bacteria are more potent than AGs administered directly to the lungs based on the exact concentrations of freely available AGs present in the lung.

Overall, the present study provides mechanistic insights into the antibacterial activity of AGs in the lung by demonstrating that: (i) Gram-negative pathogens act as a reservoir for AG antibiotics; (ii) AG-bound bacteria interact with pulmonary surfactants in the lung to achieve AG-mediated bacterial killing; and (iii) AGs originating from the Gram-negative bacterial reservoir mirror the effects of AGs administered directly to the lung. These mechanisms may explain, in part, clinical observations of AG efficacy in the lung despite the organism's *in vitro* resistance to AG antibiotics.

## Funding

This work was supported by the Cystic Fibrosis Foundation (NOTO15D0 and NOTO17Q0 to M.J.N.); the Gilead Research Scholars Program in Cystic Fibrosis Awards (to M.J.N.); and the National Institutes of Health (T32GM007347 to C.D.M.W., R00 HL143441 to L.D.P., R01 AI101171 to E.P.S., and R01 HL152210-01 to M.J.N.).

## Supplementary Material

xtac016_Supplemental_Files
