# Supplementary material for: Gram-negative bacteria act as a reservoir for aminoglycoside antibiotics that interact with host factors to enhance bacterial killing in a mouse model of pneumonia
Source: FEMS Microbes. 2022 May 13;3:xtac016. doi: 10.1093/femsmc/xtac016 (PMC9326624; doi:10.1093/femsmc/xtac016)
Supplement: xtac016_Supplemental_Files [file xtac016_supplemental_files.zip › SUPPLEMENT.docx]

**Table S1. Bacterial strains and plasmids used in this study**

| **Strain/plasmid** | **Description** | **Source** | **Mechanism of AG resistance** | **Kanamycin MIC (mg/L)** | **Gentamicin MIC (mg/L)** | **Reference** |
| --- | --- | --- | --- | --- | --- | --- |
| *A. baumannii* 17978 VU | *A. baumannii* ATCC 17978 used as wild type (WT) in experiments; Km^S^, Gm^S^ | Noto laboratory, Vanderbilt University  (Nashville, TN) | N/A | 0.9 | 0.38 (Hood-Pishchany *et al.*, 2020) | (Wijers *et al.*, 2021) |
| *A. baumannii* 17978/pMU368 | WT *Ab* 17978 transformed with pMU368 plasmid conferring kanamycin resistance |  | *aphA-3* (AG 3’-phospho-transferase) | 104.0 | 0.38 (Hood-Pishchany *et al.*, 2020) | (Hood-Pishchany *et al.*, 2020) |
| *P. aeruginosa*/pME260 | *P. aeruginosa* PAO1 transformed with pME260 plasmid conferring kanamycin resistance |  | *aph(3’)-Ia* (AG 3’-phospho-transferase) | >256 | 0.54 | This study |
| *K. pneumoniae*/pCR2.1 | *K. pneumoniae* ATCC 43816 transformed with pCR2.1 plasmid conferring kanamycin resistance |  | *aphA-3* (AG 3’-phospho-transferase) | 131.5 | 0.62 | This study |
| Tn5A7 | *Ab* 17978 UN with Tn5 inserted into putative glycosyltransferase (*lpsB*); Km^R^ |  | *aphA-3* (AG 3’-phospho-transferase) | 128 (Hood-Pishchany *et al.*, 2020) | 1.5 (Hood-Pishchany *et al.*, 2020) | (Hood *et al.*, 2013) |
| *A. baumannii* 17978 *Δhcp*::gm | *Ab* 17978 with  *hcp* replaced by gentamicin resistance cassette; Gm^R^ | Dr. M. Feldman | *aacC1* (Gm 3-N-acetyl-transferase) | 3.0 (Hood-Pishchany *et al.*, 2020) | >256 (Hood-Pishchany *et al.*, 2020) | (Weber *et al.*, 2013) |
| *E. coli* DH5⍺ | Standard laboratory strain; Km^S^, Gm^S^ | Noto laboratory, Vanderbilt University  (Nashville, TN) | N/A | 1.25 | 1.25 |  |
| *E. coli* DH5⍺/pCR2.1 | Standard laboratory strain transformed with pCR2.1 plasmid conferring kanamycin resistance | Noto laboratory, Vanderbilt University  (Nashville, TN) | *aphA-3* (AG 3’-phospho-transferase) | >256 | 0.83 |  |
| *P. aeruginosa* PAO1 | Wound isolate; Km^S^, Gm^S^ | Dr. Andrea Battistoni (Rome, Italy) | N/A | 10 (Shih & Huang, 2002) | 0.46 | (Karkhoff-Schweizer & Schweizer, 1994) |
| *K. pneumonia* ATCC 43816 | Serotype 2 strain; Km^S^, Gm^S^ | Dr. R. Stokes Peebles (Nashville, TN) | N/A | N.D. | 1.5 | (Bakker-Woudenberg *et al.*, 1985) |
| *S. aureus* USA300 LAC | Community-associated outbreak strain | Dr. Eric Skaar (Nashville, TN) | N/A | N.D. | 1.5 | (Kennedy *et al.*, 2008) |
| pCR2.1 | Cloning vector conferring kanamycin resistance |  | *aphA-3* (AG 3’-phospho-transferase) | N/A | N/A | (Spiliotis, 2012) |
| pME260 | *P. aeruginosa* general cloning vector conferring kanamycin resistance | ATCC | *aph(3’)-Ia* (AG 3’-phospho-transferase) | N/A | N/A | (Itoh & Haas, 1985) |
| pMU368 | *A. baumannii* cloning vector conferring kanamycin resistance |  | *aphA-3* (AG 3’-phospho-transferase) | N/A | N/A | (Dorsey *et al.*, 2006) |


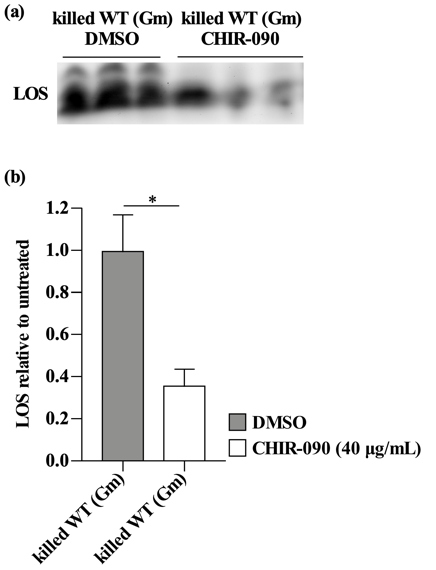


**Figure S1: Treatment of *A. baumannii* with CHIR-090 results in relative insufficiency of LOS.** (a): Gentamicin-bound, WT *A. baumannii* 17978 was treated with vehicle (DMSO) or CHIR-090 to induce LpxC inhibition as detailed in the materials and methods. LOS abundance was measured using gel electrophoresis and subsequent staining for LPS/LOS. (b): Quantification of LOS abundance in DMSO- and CHIR-090-treated, gentamicin-bound *A. baumannii* relative to DMSO-treated *A. baumannii* is depicted. (a and b): N=3 biological replicates per group, per experiment. (b): Columns depict the mean, and error bars show standard deviation of the mean. Means were compared using a Welch’s *t*-test. *: p<0.05. Gm: gentamicin.


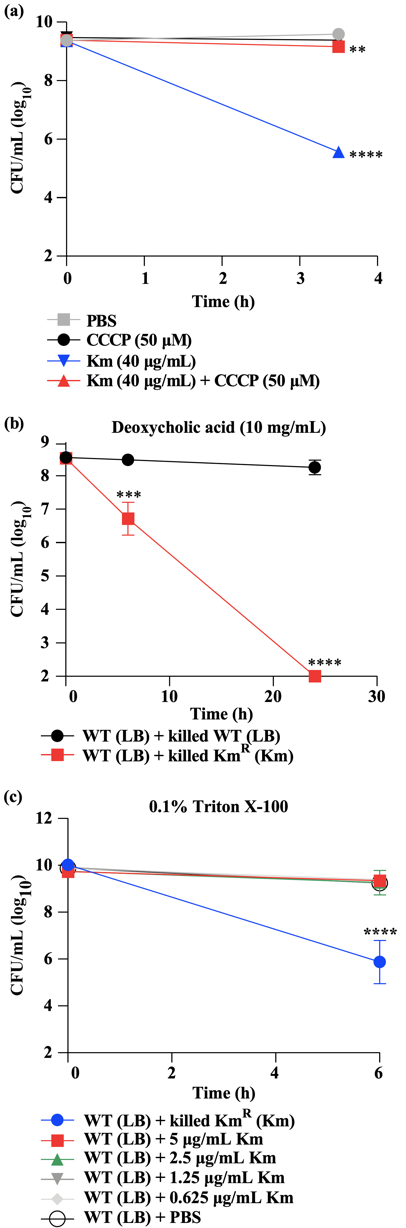


**Figure S2: *In vitro* co-incubation with kanamycin-bound bacteria and biologic detergents potentiates AG-mediated killing of AG-naïve *A. baumannii*.** (a): Mid-exponential phase, WT *A. baumannii* was incubated with PBS, CCCP, and/or kanamycin as indicated for 3.5 hours and bacterial viability was monitored over time (b): Mid-exponential phase, WT *A. baumannii* exposed to media alone (LB) was co-incubated with killed, WT *A. baumannii* exposed to media alone (LB) or killed, kanamycin-bound *A. baumannii* Tn5A7 (Km^R^) as indicated and deoxycholic acid (10 mg/mL). Bacterial viability was measured over time. (c): Mid-exponential phase, WT *A. baumannii*, grown in media alone (LB), was co-incubated with killed, kanamycin-bound *A. baumannii* Tn5A7 (Km^R^) or varying concentrations of kanamycin as indicated. Bacterial mixtures were resuspended in PBS supplemented with Triton X-100, and bacterial viability was monitored over time. (a-c): N=3 biological replicates per group, per experiment. Graphs depict representative data from two independent experiments. Symbols depict the mean and error bars show standard deviation of the mean. For each time point, means were compared using a Welch’s *t*-test (b) or one-way ANOVA adjusted for multiple comparisons (a and c). **: p<0.01; ***: p<0.001; ****: p<0.0001. Km: kanamycin.

**REFERENCES (SUPPLEMENT)**

Bakker-Woudenberg IA, van den Berg JC, Vree TB, Baars AM & Michel MF (1985) Relevance of serum protein binding of cefoxitin and cefazolin to their activities against *Klebsiella pneumoniae* pneumonia in rats. *Antimicrob Agents Chemother* **28**: 654-659.

Dorsey CW, Tomaras AP & Actis LA (2006) Sequence and organization of pMAC, an *Acinetobacter baumannii* plasmid harboring genes involved in organic peroxide resistance. *Plasmid* **56**: 112-123.

Hood MI, Becker KW, Roux CM, Dunman PM & Skaar EP (2013) genetic determinants of intrinsic colistin tolerance in *Acinetobacter baumannii*. *Infect Immun* **81**: 542-551.

Hood-Pishchany MI, Pham L, Wijers CD, Burns WJ, Boyd KL, Palmer LD, Skaar EP & Noto MJ (2020) Broad-spectrum suppression of bacterial pneumonia by aminoglycoside-propagated *Acinetobacter baumannii*. *PLoS Pathog* **16**: e1008374.

Itoh Y & Haas D (1985) Cloning vectors derived from the *Pseudomonas* plasmid pVS1. *Gene* **36**: 27-36.

Karkhoff-Schweizer RR & Schweizer HP (1994) Utilization of a mini-Dlac transposable element to create an alpha-complementation and regulated expression system for cloning in *Pseudomonas aeruginosa*. *Gene* **140**: 7-15.

Kennedy AD, Otto M, Braughton KR*, et al.* (2008) Epidemic community-associated methicillin-resistant *Staphylococcus aureus*: recent clonal expansion and diversification. *Proc Natl Acad Sci U S A* **105**: 1327-1332.

Shih PC & Huang CT (2002) Effects of quorum-sensing deficiency on *Pseudomonas aeruginosa* biofilm formation and antibiotic resistance. *J Antimicrob Chemother* **49**: 309-314.

Spiliotis M (2012) Inverse fusion PCR cloning. *PLoS One* **7**: e35407.

Weber BS, Miyata ST, Iwashkiw JA, Mortensen BL, Skaar EP, Pukatzki S & Feldman MF (2013) Genomic and functional analysis of the type VI secretion system in *Acinetobacter*. *PLoS One* **8**: e55142.

Wijers CDM, Pham L, Menon S, Boyd KL, Noel HR, Skaar EP, Gaddy JA, Palmer LD & Noto MJ (2021) Identification of Two Variants of *Acinetobacter baumannii* Strain ATCC 17978 with Distinct Genotypes and Phenotypes. *Infect Immun* **89**: e0045421.
