# Supplementary material for: Gram-negative bacteria act as a reservoir for aminoglycoside antibiotics that interact with host factors to enhance bacterial killing in a mouse model of pneumonia
Source: FEMS Microbes. 2022 May 13;3:xtac016. doi: 10.1093/femsmc/xtac016 (PMC9326624; doi:10.1093/femsmc/xtac016)
Supplement: xtac016_Supplemental_Files [file xtac016_supplemental_files.zip › RESPONSE_TO_COMMENTS FINAL.docx]

1. **RESPONSE TO REVIEWERS’ COMMENTS**

Associate Editor

Comments to the Author:

*“All reviewers found this work to be original and innovative. Several suggestions are made to experimentally strengthen the conclusions and/or to be discussed as limitations of the study in the discussion section.”*

We thank the Associate Editor for considering our manuscript and for this feedback. Based on the Reviewers’ comments, we have performed additional experiments that strengthen the conclusions of this manuscript and have expanded discussion of the limitations of this study.

Reviewer: 1

Comments to the Author:

*“Previous work from this group showed that the bacterial burden in the lungs of mice infected with WT A. baumannii could be reduced by co-inoculating with certain mutant strains of A. baumannii. They were able to determine that this was due to aminoglycoside (AG)-binding by these mutants, which led to enhanced killing in vivo. The current manuscript by Wijers and colleagues presents worked aimed at understanding the mechanism of enhanced bacterial killing mediated by AG-bound Gram-negative bacteria. The authors show that AG bind strongly to Gram-negative cells, which can affect killing of AG-naïve bacteria in vivo to as good or better extent as antibiotic treatment alone. They also perform experiments aimed at determining the site of AG binding and the components of the in vivo environment that lead to this enhanced killing by AG-bound bacteria.*

*The results [a]re intriguing and the manuscript is well written. In particular, the rationale and discussion was well thought out and presented a balanced analysis of the data.”*

We thank the Reviewer for their comments and address specific comments below.

Specific comments:

*“1) AG binding. The data showing that AGs bind to Gram negative bacteria is convincing. It is nice to see quantitative numbers here. One benefits of working with Acinetobacter is the availability of LOS- strains. Do these strains bind AGs? The binding of Gentamicin to Staph at fairly similar levels as to Acinetobacter is curious in light of the thinking that the reservoir is the OM. Or at least Gram positives may have a different reservoir. Perhaps the authors could spend a bit more space discussing this.”*

The Reviewer raises an interesting question that we have addressed experimentally. To test the hypothesis that LOS-insufficient *A. baumannii* binds and retains significantly less AGs compared to LOS-sufficient *A. baumannii* following *in vitro* exposure, LOS-insufficient *A. baumannii* was generated through pharmacological inhibition of LpxC by CHIR-090 (Barb *et al.*, 2007, Wei *et al.*, 2017). LpxC catalyzes the first committed step in lipid A synthesis (Anderson *et al.*, 1988). Treatment of *A. baumannii* 17978 with 40 μg/mL CHIR-090 resulted in a statistically significant, approximately 50%-reduction in LOS abundance as evidenced by gel electrophoresis and subsequent LPS/LOS staining (Fig. S1). Mid-exponential-phase cultures of LOS-insufficient, CHIR-090-treated *A. baumannii* were exposed to gentamicin for 3.5 hours, after which the concentration of gentamicin in bacterial cell pellets was quantified using liquid chromatography coupled with mass spectrometry (LC-MS) as described in the Materials and Methods section. Compared to vehicle-treated *A. baumannii*, the concentration of gentamicin in cell pellets of LOS-insufficient, CHIR-090-treated *A. baumannii* was reduced by approximately 50%, mirroring the reduction in LOS abundance (Fig. 3A)**.** These data further support the conclusion that anionic residues in the Gram-negative cell envelope, such as LOS or LPS, are implicated as the predominant AG binding and retention reservoir.

We agree with the Reviewer and our current hypothesis is that the sub-cellular location of the *Staphylococcus* *aureus* gentamicin reservoir is different from the Gram-negative gentamicin reservoir. This is discussed in more detail in the Discussion section (lines 436-441).

“*2) The title of the manuscript indicates synergy with host factors. Some may quibble with the use of this loaded term and the authors have not really defined it.”*

We agree with the reviewer and have replaced the word “synergize” with “interact” in the Title of the manuscript

“*3) Regarding Figure 4, why is figure 4a shown as % survival? Can this be presented in the same manner as the rest of the panel? Thus, 50% survival seems like a small decrease in the context of the Triton X-100 data later. Further, there seems to be little killing in porcine surfactant and raising questions around the suggestion that it is detergents in pulmonary surfactants that facilitate the killing. Detergents like Triton X-100 seem to be able to enhance killing, but this doesn’t seem to be happening with porcine surfactant. In previous work (Hood-Pischany 2020), SDS did not have any effect in similar experiments. Can the authors comment on this. Is there a particular property of the detergent that is necessary for enhancing killing?”*

We have revised Fig. 4A such that the data of all panels in this figure are now represented in a similar manner (i.e. CFU/mL (log_10_) over time). In the presence of the detergents Triton X-100 (0.1%) or deoxycholic acid (10 mg/mL), there was a >3-log_10_ reduction in the survival of AG-naïve *A. baumannii* after co-incubation with kanamycin-bound *A. baumannii* for at least 6 hours (Fig. 4E and F; Fig. S2B and C), and this magnitude of reduced survival is not fully recapitulated by incubation with porcine surfactant for 6 or 24 hours (data not shown). The porcine surfactant used in this study (Curosurf) is bronchoalveolar lavage fluid obtained by porcine lung lavage with 0.9% sodium chloride solution. Therefore, the porcine surfactant is significantly diluted by the saline solution and the resulting detergent suspension is likely far less concentrated than the Triton X-100 or deoxycholic acid solutions used in this study, and less concentrated than what is encountered inside the murine lung (lines 444-448).

As pointed out by the Reviewer, the characteristics of the detergent impact bacterial killing in the presence of AGs. In contrast to the non-ionic detergent Triton X-100, SDS is anionic. This suggests that anionic detergents do not interact with AG-naïve bacteria to enhance their AG-mediated killing. We hypothesize that anionic detergents have reduced ability to bind to the anionic Gram-negative OM relative to non-ionic detergents, which may prevent the interactions with AGs that result in bacterial killing. The Discussion has been revised to include these comments (lines 454-458).

Reviewer: 2

Comments to the Author:

*“The manuscript from Wijers et al describes an interesting phenomenon whereby aminoglycoside antibiotics bind to bacteria and can then go on to kill other bacteria only in the context of the lung environment. This work builds on previous findings to provide additional insight, in particular the potential role of lung surfactant in liberating aminoglycosides from exposed bacteria, which can then go on to kill previously non-exposed bacteria. The manuscript is well written and it’s clear why experiments were done and how one leads to the next. The methods used are appropriate and experiments are well controlled.*

*My only issue is that it would’ve been easy for the authors to test their model by showing that lung surfactant liberates aminoglycosides from exposed bacteria (decrease in [AG] in cell pellet and presence of AG in supernatant containing surfactant).”*

We thank the Reviewer for their comments and agree with the suggestion for additional experimentation. To test the hypothesis that detergents liberate AGs from AG-bound bacteria, gentamicin-susceptible and gentamicin-resistant *A. baumannii* were exposed to gentamicin for 3.5 hours, chemically killed, resuspended in PBS with or without deoxycholic acid, and incubated at 37 ℃ for 6 hours with constant agitation. Following incubation, the concentration of gentamicin in bacterial cell pellets was quantified using LC-MS. The concentration of gentamicin in the supernatants could not be quantified as the presence of detergent interferes with LC-MS analysis. In comparison to incubation in PBS alone, incubation in PBS supplemented with deoxycholic acid did not significantly alter the concentration of gentamicin in cell pellets of gentamicin-bound bacteria for both gentamicin-susceptible and gentamicin-resistant *A. baumannii* (Fig. 4G). These data do not support the conclusion that detergents liberate AG molecules from AG-exposed bacteria. Instead, detergents may increase susceptibility of AG-naïve, co-infecting bacteria to AG antibiotics by increasing cell membrane permeability (Radlinski *et al.*, 2019). These comments have been included in the revised manuscript (lines 448-455).

Reviewer: 3

Comments to the Author:

*“This was an interesting paper that investigates the ability and mechanism for aminoglycoside (AG) antibiotics to associate with Gram-negative bacteria, and subsequently for AG-bound bacterial cells to evoke an antibacterial effect against naïve bacterial cells in vivo (in the lung) but not in vitro. This is a curious phenomenon, and we found the work to be interesting and fairly comprehensive – but with some important limitations. We believe that the investigators have demonstrated an interaction between AGs and bacterial cells; however, we do not feel the studies support the full extent of their conclusions. Ideally, additional experiments would be undertaken and included in the manuscript to address the limitations we noted (see below). If that is not possible, the limitation points should be highlighted in the Discussion as substantial drawbacks to the research and aims for further study.”*

We thank the Reviewer for their comments and we have performed additional experiments to strengthen the conclusions of this manuscript. In addition, we have revised the Discussion section of the manuscript to expand on the study limitations, including those mentioned by the Reviewers.

Scientific limitations:

*“1. Drug concentrations were only measured in cell pellets instead of different fractionated components. We recommend that concentrations be measured in cell lysates as well (particularly after extracellular AG is displaced from the OM) to better understand where the drug is located; also, if drug can be displaced from the OM after washing, it would be beneficial to measure conc in the supernatant.”*

We have performed an experiment to test the hypothesis that detergents liberate AGs from AG-bound bacteria that includes the determination of cell lysate AG concentrations (see Reviewer 2 “Comments to the Author”). These data do not support the conclusion that detergents displace AGs from the bacterial AG reservoir into the supernatant (Fig 4G). However, the concentration of AGs in the supernatant could not be measured directly as detergents would interfere with the LC-MS analysis. Further, following incubation with detergent (deoxycholic acid) or vehicle (PBS), gentamicin-bound *A. baumannii* cells were lysed, and the concentration of gentamicin in soluble bacterial lysates was measured using LC-MS as described in the Materials and Methods section. The concentrations of gentamicin detected in soluble bacterial lysates were approximately 10% of those detected in cell pellets of chemically killed bacteria for both gentamicin-susceptible and gentamicin-resistant *A. baumannii* (Fig 4G). To generate soluble bacterial lysates, both the original culture supernatant and the cell debris were separated from the lysate fraction. Therefore, it is possible that treatment with detergent differentially displaced AG to either the culture supernatant or cell debris fractions, in comparison to treatment with vehicle (PBS). Taken together, these data further support the conclusion that the bacterial cytoplasm does not serve as a major AG binding and retention reservoir.

*“2. Correlations are not provided between bacterial killing and drug concentrations, particularly in the context of the different MICs for the strains tested. Based on the data shown in Figure 1, bacteria pre-incubated with AGs appear to retain very high drug conc (>50 ug/mL); this alone could explain much of the data, especially if detergent is simply displacing AGs from the OM.”*

Throughout the present study, several strains of AG-bound bacteria are used to co-inoculate mice at the time of infection with live, AG-naïve bacteria. *A. baumannii* 17978 VU was used as the live, AG-naïve inoculum (i.e. “the tested strain”) for all animal infections and *in vitro* experiments included in this study. The kanamycin and gentamicin MICs for this strain are 0.9 mg/L and 0.38 mg/L, respectively (Table S1). Therefore, AG-bound bacteria likely retain ample antibiotics to overcome the MIC of “the tested strain” in the experiments included in this study. Of note, the additional experiments do not support the conclusion that detergents displace AGs from AG-bound bacteria. Instead, detergents may increase susceptibility of AG-naïve, co-infecting bacteria to AG antibiotics by increasing cell membrane permeability (see Reviewer 2 “Comments to the Author”).

Further, *in vitro* susceptibility of “the tested strain” to AGs is not required for enhanced bacterial killing inside the murine lung mediated by AG-bound bacteria. We have previously demonstrated that co-inoculation of mice with kanamycin-bound bacteria at the time of infection with live, kanamycin-resistant *A. baumannii* AB5075 significantly increased killing of *A. baumannii* AB5075 compared to co-inoculation with bacteria unexposed to any antibiotics (Hood-Pishchany *et al.*, 2020). Since the kanamycin MIC for *A. baumannii* AB5075 is >256 mg/L, the presence of a bacterial AG reservoir large enough to overcome the MIC of the co-infecting strain alone does not explain the phenotype observed in this study. In the revised manuscript, the Discussion section has been expanded to include these comments (lines 395-397, 448-455, and 476-477).

*“3. Drug conc were measured in lung homogenate instead of epithelial lining fluid (ELF). The investigators cannot make any meaningful conclusions from these results as tissue homogenate data is unreliable; see Mouton 2007 ‘Tissue concentrations: do we ever learn?’”*

According to Mouton and colleagues (2007), “the use and determination of tissue concentrations derived from homogenates to draw direct conclusions on drug activity are unwarranted and/or unreliable. Statements that infer activity of the drug based on concentrations measured in relation to the susceptibility (MIC) of the agent should be avoided” (Mouton *et al.*, 2008). We agree with this statement, and we deliberately have not concluded that the drug concentrations depicted in Fig. 2D are sufficient to overcome the MIC of infecting bacteria. We merely argue that the bacterial burdens of mice co-inoculated with gentamicin-bound bacteria are lower than the bacterial burdens of mice co-inoculated with gentamicin solution, even though more gentamicin is detected in lung homogenates of the latter group of mice. Of note, Mouton and colleagues (2007) also state that: “If a compound is distributed mainly extracellularly, such as […] aminoglycosides, grinding up the tissue means dilution of the drug by mixing intracellular and extracellular fluids, resulting in underestimation of its concentrations at the site of infection” (Mouton *et al.*, 2008). Therefore, the gentamicin concentrations depicted in Fig. 2D may underestimate the concentrations of gentamicin present in the ELF, as mice were challenged with gentamicin solution or gentamicin-bound bacteria intranasally. We have revised the Discussion section to include the comments described above, and have replaced the phrase “pulmonary AG concentrations” with “AG concentrations in lung homogenates” to more accurately reflect the data described above (lines 463-467 and 506-514).

*“4. Authors refer several times to the unexpected finding that inhaled AGs work better in clinical pneumonia than other indications. This is likely due to the fact that very high drug conc can be achieved locally in the lung with inhaled delivery, yet this simple PK/PD concept is not discussed anywhere in the manuscript.”*

We agree with the reviewer that treatment with inhaled AG antibiotics leads to higher local drug concentrations inside the lungs compared to systemic treatment. We have revised the Introduction and Discussion sections to include this concept (lines 74-79 and 474-477).

Overall suggestions to improve the reader’s experience:

*“1. The manuscript was generally hard to follow/understand even though the material is not particularly difficult. Suggest refining the text with a focus on greater clarity.”*

We have revised all sections of the manuscript to improve clarity.

*“2. Introduction is inadequate; all relevant concepts should be introduced here instead of in the Results section (e.g. how AGs interact with bacterial cells and membranes, how AGs differ from other antibiotics in these interactions, the role of surfactant, etc.)”*

We agree and have revised the Introduction section to include details on the mechanism of action of AG antibiotics, including how they interact with bacterial membranes. Details on the role of pulmonary surfactant have been included as well (lines 50-63 and 81-88).

*“3. Too many method details in Results section and Figure legends; move these details to Materials*

*and Methods instead.”*

We have revised the Materials and Methods and Results sections, as well as the Figure legends, to move details on experimental methods to the Materials and Methods section.

*“4. Some figures are difficult to interpret, especially when multiple notations are provided along the*

*x-axis (and in different orientations, i.e. some text diagonal and some text horizontal).*

Figures have been revised to improve interpretability. For example, Figures 1 and 2 have been revised such that the orientation of x-axis annotations is identical for all panels.

*“5. Strain names are rarely provided in the text and only in some (but not all) of the figures. MICs are not provided at all (except in the Supplementary table). These are needed for context, and all results should include commentary on how MICs for the strains tested compare to the measured drug conc.”*

To improve clarity and readability, many strain names have been omitted from the text of the Results section and have been included in the Figure legends instead. In the revised manuscript, care has been taken to include all relevant strain names in the appropriate Figure legends as well as the Materials and Methods section. In addition, relevant MICs (e.g. kanamycin or gentamicin) for each strain have been included in the Figure legends.

Line comments:

*“1. Title (and throughout text): words and phrases like “synergize with host factors” and “interact” and “collaborate” imply an active cooperation between the AG-bound bacteria and the host innate or adaptive immune response, which seems to be an overstatement. The findings here appear to indicate a more passive event whereby the presence of surfactant in the lung displaces AG from the OM.”*

We agree with the Reviewer that terms including “synergize” and “collaborate” may be inaccurate, as the phenomena described in this manuscript may indeed be passive. Because the term “interaction” is more general and includes passive interactions, we have opted for the use of this term in the title as well as throughout the revised manuscript (Reviewer 3 “Comments to the Author”).

*“2. Abstract (and throughout text): “Gram-negative bacteria” and “aminoglycosides” are used broadly throughout; however, not all Gram-negative bacteria or AGs have been tested.”*

In agreement with the Reviewer, we have revised the manuscript to more accurately reflect the data. The phrase “Gram-negative bacteria” has been replaced with “several Gram-negative bacteria” or the specific species/strain tested, and “aminoglycosides” has been replaced with kanamycin and/or gentamicin as appropriate (e.g. lines 30, 35, and throughout).

“3. Abstract, line 45: how might this finding expand clinical utility, as AGs are already commonly used for lung infections (especially inhaled).”

The use of inhaled AGs is occasionally suggested for the treatment of hospital-acquired or ventilator-associated pneumonia (HAP or VAP, respectively) caused by multi-drug resistant (MDR) Gram-negative pathogens that are susceptible to AG antibiotics (Kalil *et al.*, 2016, Leone *et al.*, 2018). By contrast, treatment with systemically administered AG antibiotics is not recommended for HAP or VAP in part because of poor lung penetration (Panidis *et al.*, 2005, Kalil *et al.*, 2016). Therefore, AGs administered directly to the lungs appear to be uniquely effective likely in part because of high local drug concentrations that are achieved in this manner. We reason that the present study provides additional insights into why AG antibiotics may be more potent inside the lung as these data indicate that the presence of pulmonary surfactant increases AG-mediated bacterial killing. In addition, previous work by our group has demonstrated that treatment with kanamycin-bound bacteria may be effective even if the infecting bacteria demonstrate *in vitro* resistance to kanamycin (Hood-Pishchany *et al.*, 2020). This is consistent with studies demonstrating that treatment with inhaled tobramycin may be beneficial even if infecting isolates demonstrate *in vitro* resistance to tobramycin (Ramsey *et al.*, 1999). Therefore, mechanistic observations from this study may inform future clinical studies testing the efficacy of inhaled AG antibiotics for Gram-negative lung infections, which may expand their utility to treatment of pneumonia caused by bacteria with *in vitro* resistance to AGs. In the revised manuscript, the Discussion section has been expanded to include the comments above (lines 491-499).

*“4. Line 63: what is known about AG conc in tissues after systemic delivery vs. inhaled? Likely AG conc in plasma after IV dosing do not reach high enough conc in tissues (without also causing toxicity) to effectively treat infection. Note that “bacteremia” is not an infection in the blood per se, but it is generally seeded from an infection located elsewhere; therefore, you cannot cure the bacteremia without also curing the ‘hidden’ infection (which can be more difficult if drug does not easily reach the site).”*

Systemically administered AG antibiotics have poor lung penetration, requiring peak serum concentrations in order to achieve biologically active concentrations inside the lungs (Panidis *et al.*, 2005, Boselli *et al.*, 2007). Indeed, this increases the risk of ototoxicity and nephrotoxicity, two serious side effects associated with AG use (Mingeot-Leclercq & Tulkens, 1999, Dobie *et al.*, 2006). Therefore, systemically administered AG antibiotics are not recommended for the treatment of HAP or VAP. The Introduction and Discussion sections have been revised to include these comments (lines 77-79 and 474-487).

*“5. Line 65: it seems overstated to say that “…alterations to the bacterium that interact with host factors to achieve broad-spectrum suppression of pneumonia…” It does not appear that it is changes to the bacterial cell, nor does it appear to be ‘interactions’ with host factors. Also, what is meant by “broad spectrum?” Usually that term is used to refer to antibiotics which have activity against a wide range of bacteria, including Gram-pos[i]tive and Gram-negative. Suggest choosing an alternate word here.”*

Based on our previous work, the hypothesis at the outset of these studies was that “exposure of the human pathogen *Acinetobacter baumannii* to an AG antibiotic *in vitro* causes alterations to the bacterium that interact with host factors to …” (Hood-Pishchany *et al.*, 2020). Because the present study was undertaken to test this working hypothesis, it was included as such in the current manuscript. We reason that the binding and retention of AG antibiotics by Gram-negative bacteria does constitute an alteration to the bacterial cell, especially as ionic interactions between the Gram-negative OM and the polycationic AG antibiotic molecules strong enough to withstand multiple washes are implicated. We agree with the Reviewer that the use of “collaborate” or “synergize” is an overstatement and have changed these terms to “interaction”.

In the previous work referenced above, co-inoculation of mice with AG-exposed *A. baumannii* increased killing of several Gram-negative pathogens inside the murine lung, including *A. baumannii*, *Klebsiella pneumoniae*, and *Pseudomonas aeruginosa*. As described in that publication, these data suggest that co-inoculation with AG-exposed *A. baumannii* has activity against multiple Gram-negative pathogens (Hood-Pishchany *et al.*, 2020). In agreement with the Reviewer, the term “broad-spectrum” has been omitted to indicate that AG-bound bacteria have activity against multiple Gram-negative pathogens, specifically (lines 89-91).

*“6. Line 68: same comment about “interaction” between bacteria and host”*

Please see our response to Reviewer 3 Line Comments 1 and 5.

*“7. Lines 69-70: can you suggest what the implications might be? AGs are already used for lung infections, particularly as inhaled delivery.”*

The Introduction and Discussion sections have been revised to specify what these implications might be (also, see our response to Reviewer 3 Line Comment 3).

*“8. Materials and Methods section: there is little context for what is being done and why; can you please explain what the different assays are for and what they are intended to show? (Some of this is in the Results section; however, it probably does not belong there and should be moved to this section instead.)”*

The Materials and Methods section has been revised to include more context for each experiment (lines 99-238).

*“9. Line 81: why were 40 and 50 ug/mL chosen as the conc for Km and Gm, respectively? Are they clinically relevant?”*

The antibiotic concentrations listed above were used as these are standard for propagating kanamycin-resistant or gentamicin-resistant bacteria on selection media. The initial observation that co-inoculation with AG-exposed bacteria significantly reduces the burdens of co-infecting, AG-naïve bacteria inside the murine lung was made using bacteria that were grown in media supplemented with kanamycin for the maintenance of a transposon insertion (Hood-Pishchany *et al.*, 2020). Therefore, unless stated otherwise, these concentrations were used for all subsequent experiments as well.

*“10. Lines 84-85: can you confirm that this method of chemically killing cells has no effect on cell integrity?”*

Ethanol causes membrane damage and denaturation of proteins, whereas acetone increases membrane fluidity (McDonnell & Russell, 1999, Dyrda *et al.*, 2019) (lines 116-118). To the authors’ knowledge, chemical killing of bacteria using an ethanol/acetone mixture does not lyse the cells.

*“11. Murine infection studies: were the animals randomized? If so, how? And if not, why not? Were the animal studies blinded? If so, how? And if not, why not? How did you determine the number of animals required per group (i.e. were power calculations conducted pre-study, and what were they powered for)?”*

Animals were randomly assigned to experimental groups using a GraphPad Prism random number calculator (<https://www.graphpad.com/quickcalcs/randomize1/>). Animals, and the samples derived from them, were identified by a combination of cage and animal numbers only. Therefore, at the time of harvest, the experimenter was blinded to experimental group assignment. Prior to animal experiments, power calculations were performed and powered for a 4-log_10_ difference in bacterial burden with an estimated standard deviation of 2-log_10_ and an ⍺ of 0.05. The Materials and Methods section has been revised to include these comments (lines 233-236).

*“12. Murine infection studies: Please refer to the ARRIVE Guidelines (https://arriveguidelines.org) to ensure all relevant animal-related parameters are included”*

The revised manuscript includes all relevant animal-related parameters per the referenced ARRIVE guidelines, including study design; sample size (indicated in each figure legend); randomization; experimental procedures; statistical methods; and results.

*“13. Line 97: were mice anesthetized for the infection? If so, what method/route/dose of anesthesia was used?”*

Prior to infection, mice were anesthetized with 250-450 μL of a 2,2,2-tribromoethanol solution (25 mg/mL) via intraperitoneal injection. Adequate anesthesia was assessed for each animal by observing the absence of limb movement in response to applying pressure on the toe pads of both hind limbs. In the revised manuscript, this information has been included in the Materials and Methods section (lines 139-141).

*“14. Line 102: how do you ensure the inoculum is consistent in each group, especially if the cells have undergone different pre-treatments? Do you use ODs to estimate densities of the suspensions? Did you actually measure the bacterial load in the inocula in each study to ensure they were consistent across all the groups?”*

Prior to infection, target bacterial densities were estimated by measuring the optical density at 600 nm (OD_600_) of each bacterial suspension. Following infection, inoculums for each group were serially diluted in PBS and plated on LBA for bacterial enumeration to verify that they were consistent across all groups as well as with target inoculums. The Materials and Methods section has been revised to include this information (lines 154-155).

*“15. Line 104: how were lungs homogenized?”*

Lungs were submerged in 500 μL of sterile, ice-cold PBS and homogenized with stainless steel beads in a benchtop bead beater. This information has been included in the revised manuscript (lines 155-158).

*“16. Lines 109-110: please provide more detail for how mouse lung homogenates were processed, i.e. was the tissue completely disrupted using a bead beater or other method? (As noted in the Limitations above, lung concentrations should be measured in ELF by performing a bronchoalveolar lavage and then centrifuging to remove cells; the supernatant represents ELF and should be measured for drug conc using urea to normalize for the lavage dilution factor. See Rodvold 2011 “Penetration of anti-infective agents into pulmonary epithelial lining fluid” for further info.)”*

Lung tissue was completely disrupted in a bead beater using stainless steel beads (also, see our responses to Reviewer 3 Line Comment 15 and Reviewer 3 “Scientific Limitation” 3).

*“17. Lines 110-111: why was an ELISA used to measure conc, especially when LC/MS methods were*

*also used?”*

The discovery that Gram-negative bacteria bind and retain kanamycin and gentamicin at high concentrations following *in vitro* exposure was surprising. To corroborate the results obtained using ELISAs, LC-MS was used as a secondary method.

*“18. Line 136: suggest different wording other than “collaborate with host factors”*

See our response to Reviewer 3 Line Comment 1.

*“19. Line 140: how can you incubate sensitive strains with AGs and have them survive? Have you investigated whether this method ‘weakens’ the cells in any way, making them less virulent and/or more susceptible to host factors or future antibiotic exposure?”*

AG-susceptible strains were grown in media (LB) without antibiotics until mid-exponential phase. Mid-exponential-phase bacteria were incubated with kanamycin or gentamicin for an additional 3.5 hours as detailed in the Materials and Methods section (lines 109-112). This leads to a significant decrease in bacterial survival (Fig 3B and D; Fig. S2A). Following incubation with kanamycin or gentamicin, bacteria were killed, washed, and diluted as described in the Materials and Methods section. We thank the Reviewer for the interesting experimental suggestions. We agree that it would be interesting to investigate whether AG-susceptible bacteria that survive AG exposure are more susceptible to future antibiotic exposure, for example. However, we reason that this is outside the scope of the present manuscript.

*“20. Line 147: you may be detecting low conc of Km in S. aureus cell pellets because the washing is incomplete; consider using a higher volume for the washing steps (e.g. 10x more media) to further dilute out the drug”*

We agree with the Reviewer’s assessment and have revised the manuscript to include this comment (lines 434-435).

*“21. Line 168: please explain why both ELISA and LC/MS methods were used (similar to the comment for Lines 110-111)”*

Please see our response to Reviewer 3 Line Comment 17.

“22. Lines 176-177: mice dosed IN with Gm or PBS were only those given AG-naïve bugs, correct? Please clarify”

For this experiment, mice were co-inoculated with live, AG-naïve WT *A. baumannii* 17978 and killed *A. baumannii ∆hcp*::gm grown without (Fig. 2C, groups 1 and 3) or with (Fig. 2C, group 2) gentamicin as indicated. All mice received a second inoculum of either PBS (Fig. 2C, groups 1 and 2) or gentamicin in PBS (Fig. 2C, group 3). As such, one group of mice that was dosed intranasally with PBS received AG-bound bacteria (Fig. 2C, group 2). This is in contrast to a different group of mice that was dosed intranasally with gentamicin in PBS and received AG-naïve bacteria only (Fig. 2C, group 3). The manuscript has been revised to include these clarifying descriptions (lines 151-155)

“23. Line 179 (and elsewhere): what is the 0 timepoint?”

For the animal infection, the 0 timepoint refers to mice that were euthanized and whose lungs were harvested immediately after intranasal challenge. For *in vitro* experiments, the 0 timepoint refers to bacterial viability measurements taken immediately prior to incubation at 37 ℃, after reagents such as detergents or surfactant proteins were added to the samples.

*“24. Line 180 (and elsewhere): provide the statistical tests used and the resulting p-values for statements saying “significantly lower/higher”*

General descriptions of statistical analyses (e.g. p values of comparisons that are considered statistically significant) are included in the Materials and Methods section (lines 236-238). Specifics on which statistical tests were used for each experiment, as well as the degree of statistical significance for each comparison, are included in the figure legends.

*“25. Line 183: please qualify the statement here; Gm was present in the lung, but we do not know where it was located within the tissue or whether it was unbound and freely available to exert its effect on the bacteria”*

We agree with the Reviewer that based on this experiment it cannot be determined where within the lungs the gentamicin was present. Based in part on the reference provided by the Reviewer, we reason that the most likely location is in the distal airways and alveolar spaces, as AG antibiotics are predominately distributed extracellularly (Mouton *et al.*, 2008). The Discussion section has been revised to include these qualifying comments (also, see our response to Reviewer 3 “Scientific Limitation” 3).

*“26. Lines 191-192: while we agree that this is the likely mechanism, suggest re-wording this statement as it has not been proven”*

We agree with the Reviewer that this has not been proven and have reworded this statement accordingly (line 298-299).

*“27. Line 201: Do AGs bind to the OM before being internalized? If so, why doesn’t all the AG bound outside the cell ultimately end up inside the cell? What causes the AG to just stay bound on the cell’s surface?”*

As detailed in the Introduction, the first step of the mechanism of action of AG antibiotics is the binding of polycationic AG molecules to anionic residues on bacterial cell envelopes. AG internalization into the bacterial cytosol is comprised of two energy-dependent steps (see Introduction). Because these steps are energy-dependent, it may be energetically more favorable for AG molecules to remain bound to the cell envelope. It was recently demonstrated that AGs bind bacterial membranes in a saturable fashion (Sabeti Azad *et al.*, 2020). Therefore, we hypothesize that for every AG molecule that is internalized into the bacterial cytosol, it is readily replaced by a new AG molecule from the extracellular environment, as long as free AG molecules are available in the media. This is supported by data demonstrating that the concentration of gentamicin in lysates of gentamicin-exposed *A. baumannii* is a fraction of that detected in bacterial cell pellets (Fig. 4G).

*“28. Lines 206-207: we feel that the experiments used to conclude this are not comprehensive enough; for example, can you measure how much AG is internalized vs. bound to the surface? See the Limitations noted above.”*

We have performed additional experiments to further support the conclusions of this manuscript. First, LOS-insufficient *A. baumannii* retained significantly less gentamicin than LOS-sufficient *A. baumannii*, suggesting that inhibiting AG binding to the bacterial OM decreases the size of the Gram-negative AG reservoir (Fig 3A and our response to Reviewer 1 “Specific Comment” 1) (lines 301-308). In addition, the concentration of gentamicin detected in lysates of gentamicin-bound *A. baumannii* was approximately 90% lower than the concentration of gentamicin detected in bacterial cell pellets (Fig. 4G and our response to Reviewer 3 “Scientific Limitation” 1) (lines 379-386). We reason that the results of these experiments provide additional evidence that the Gram-negative OM is implicated as the predominant AG reservoir.

*“29. Line 271: suggest a word other than “indicate” as we feel the data is suggestive but not definitive”*

In the revised manuscript, the word “indicate” has been replaced by “suggest”.

*“30. Line 222: why were these concentrations chosen? Are they clinically relevant?”*

The highest concentration (40 μg/mL) was chosen as this is standard for propagating kanamycin-resistant bacteria on selection media (see our response to Reviewer 3 Line Comment 9).

*“31. Lines 226-227: should not state that the quantity determines killing without actually measuring the respective quantities; how much drug was present after incubating in 10 ug/mL vs. 40 ug/mL? Enough to cover the MIC?”*

We agree with the Reviewer and have reworded this statement to more accurately reflect the data (lines 338-339).

*“32. Lines 227-229: what about other resistance mechanisms? Are there any other relevant mechanisms, especially those that affect the OM (e.g. decreased binding, permeability and/or increased efflux, etc.)?”*

Other mechanisms of AG resistance include ribosome modification and LPS modification to impede AG binding to target ribosomes or the bacterial cell envelope, respectively (Yokoyama *et al.*, 2003, Kwon & Lu, 2006, Wachino & Arakawa, 2012, Garneau-Tsodikova & Labby, 2016). Although no strains with such mechanisms of AG resistance were used in this study, we hypothesize that bacteria in which AG resistance is conferred by LPS/LOS modification bind and retain significantly less AGs following *in vitro* exposure. This is supported by our data obtained with LOS-insufficient *A. baumannii* (Fig. 3A and our response to Reviewer 1 “Specific Comment” 1).

*“33. Line 247: how were the conc of SP-B and SP-D chosen? Are they biologically relevant conc?”*

These concentrations were based on a previous report demonstrating that surfactant proteins have antibacterial activity at concentrations of both 5 and 25 μg/mL (Wu *et al.*, 2003).

*“34. Lines 256-257: you should be able to measure the amount of free Km in the supernatant to show that Triton X simply displaces AG from the OM”*

We have performed additional experiments to test the hypothesis that detergents displace AGs from AG-bound bacteria (Fig. 4G and our response to Reviewer 2 “Comments to the Author”).

*“35. Lines 264-266: What is meant by “increased killing?” If the release of AGs within the lung simply raises local drug conc, then the AGs aren’t ‘increasing killing’ per se; the bacteria are acting like a drug delivery system, and the AGs are behaving normally (i.e. killing bugs via their normal MoA) but doing it better because AG conc are higher.”*

We agree with the Reviewer and have reworded this statement accordingly (line 343 and throughout).

*“36. Line 273: what is already known about the mechanism of AG post-antibiotic effects?”*

The post-antibiotic effect of AGs describes the phenomenon of continued AG-mediated bacterial killing in a dose-dependent manner after removal of antibiotics from the medium (Isaksson *et al.*, 1988). Anionic sites on bacterial cell envelopes (e.g. LPS) have been implicated as the binding site of AG molecules responsible for the post-antibiotic effect (Jackson *et al.*, 1990). Congruent with evidence provided in the present manuscript, these findings suggest that AGs remain bound to bacterial membranes after bacteria are resuspended in AG-free media. Membrane-bound AGs then enter the bacterial cytosol in an energy-dependent manner (Jackson *et al.*, 1990), bind to bacterial ribosomes, and exert their bactericidal effects. In the revised manuscript, the discussion on the post-antibiotic effect of AG antibiotics has been expanded (lines 410-414)

*“37. Lines 294-296: Yes, that is possible, but also consider that detergents are simply disrupting the AG-OM binding and causing release of drug into the environment, thus making it available to exert its antibacterial effect*”

We have performed additional experiments to test the hypothesis that detergents displace AGs from AG-bound bacteria (Fig. 4G and our response to Reviewer 2 “Comments to the Author”).

*“38. Lines 299-301: this cannot reliably be concluded from lung homogenate data”*

We have reworded this statement to more accurately reflect the data. In addition, we have included the limitations of lung homogenate AG concentration data in the Discussion setting (lines 506-514). Also, see our response to Reviewer 3 “Scientific Limitation” 3.

*“39. Line 305-306: as noted above, please review and address the more likely reason (i.e. that inhaled delivery of antibiotics creates very high localized drug conc in the lung, thus enabling them to kill bacteria even with high MICs)”*

The possibility of high, localized AG concentrations achieved by challenging mice intranasally with AG-bound bacteria has been included in the Discussion section (lines 394-398; 476-477; and 493-494)

*“40. Lines 307-308: review and address if AGs have lower urinary excretion than the other agents mentioned; this would likely explain their reduced efficacy in UTI”*

Review of the literature indicates that AG antibiotics are excreted into the urine at high concentrations and rapidly exceed plasma concentrations after parenteral administration (Naber *et al.*, 1973, Wood & Farrell, 1976). These findings do not suggest that low urinary excretion of AG antibiotics is responsible for the observed increase in bacteriological failure at the end of treatment in comparison to other antibiotics (Vidal *et al.*, 2007) (lines 477-481).

*“41. Lines 314-316: consider the high local drug conc from inhaled treatment”*

Please see our response to Reviewer 3 Line Comment 39.

*“42. Lines 319-320: maybe, but high local drug conc after inhaled treatment makes more sense”*

Please see our response to Reviewer 3 Line Comment 39.

*“43. Lines 321-322: we would really like to know how much free drug is present in the ELF after coinfecting*

*with AG-bound bacteria. Based on Figure 1, it seems that the bacterial cells hold enough drug on the OM that collectively (with a 3x10e8 inoculum) a local drug conc of >40 ug/mL could easily be achieved in the lung if most of the drug is released by surfactant”*

We agree with the Reviewer that the data presented in this manuscript suggest that intranasal administration of AG-bound bacteria constitute a highly efficient drug delivery system for AG antibiotics. We reason that obtaining precise AG drug concentration measurements of the murine epithelial lining fluid is outside the scope of the present manuscript. Please, also see our response to Reviewer 3 “Scientific Limitation” 3.

*“44. Lines 333-334: we do not agree with this statement; presumably, it is based on lung homogenate data, which is unreliable”*

We agree with the Reviewer and have revised this statement accordingly (lines 517-518).

*“45. Figure 1: please use the same units for all drug conc graphs; units of ug/mL are preferred so we can easily compare them to strain MICs”*

We have revised the figures such that all graphs display AG drug concentrations in units of μg/mL.

*“46. Figure 2c: with the high amounts of AG in the lung homogenates, how are you ensuring that the bacterial counts are not affected by ex vivo killing on the plates?”*

As demonstrated in this manuscript, in the absence of pulmonary surfactant (components), *in vitro* co-incubation with AG-bound bacteria does not negatively affect AG-naïve *A. baumannii* viability (Fig. 1D). In theory, residual pulmonary surfactant present in lung homogenates could facilitate *ex-vivo*, AG-mediated bacterial killing on LBA plates. However, we reason that the potential impact of this on the data depicted in Fig. 2C is limited. First, pulmonary surfactant present in the murine lung is diluted through the process of generating lung homogenates (see Materials and Methods section) and then again through serial dilution in PBS and plating on LBA for bacterial enumeration. Second, lung homogenate gentamicin concentrations are significantly higher in mice treated with gentamicin solution in comparison to mice treated with gentamicin-bound bacteria, despite significantly greater bacterial burdens in the former group of mice. *Ex-vivo*, gentamicin-mediated killing would be expected to bias the data in the opposite direction.

*“47. Figure 2d: units should be ug/g tissue; however, if done with ELF, then it should be ug/mL corrected for dilution using the urea method”*

We agree and have revised Figure 2D such that gentamicin concentration is now represented as μg/g of lung tissue.

*“48. Figure 3c: should there be a PBS control?”*

The experiment depicted in Fig. 3C was aimed at testing the hypothesis that kanamycin-mediated killing of susceptible *E. coli* can be prevented by adding MgSO_4_ or CCCP to the media at the time of treatment with kanamycin. By comparing data to a control comprised of bacteria treated with kanamycin only, we reason that this hypothesis can be tested adequately in the absence of a PBS control.

*“49. Figure 3 legend, Line 552-553: this was an in vitro assay in Fig 3c?”*

The data depicted in Fig. 3D represent *in vitro* survival data of mid-exponential-phase cultures of *E. coli* exposed to kanamycin and treated with or without MgSO_4_ or CCCP as indicated. The figure legend has been revised to clarify this (lines 774-777).

*“50. Figure 4a: why was % survival used here and not actual CFU counts? Please describe (in the Methods) how % survival was calculated.”*

Fig. 4A has been revised to depict the density of viable bacteria in CFU/mL (log_10_). Please, also see our response to Reviewer 1 “Specific Comment” 1.

*“51. Figure 4f: what conc of Km were the killed bacteria incubated in?”*

As detailed in the Materials and Methods section, bacteria were incubated in media supplemented with 40 μg/mL for all relevant experiments unless stated otherwise. Of note, for all experiments in the present study, chemically killed bacteria were incubated in the presence of AG antibiotics (kanamycin, gentamicin) prior to chemical killing.

*“52. Schematic on page 27: please confirm vs. methods described in the text as we thought all bacteria were first grown in LB, and then only exposed to AG for the last 3.5h growth phase”*

AG-resistant bacteria were grown to exponential phase in the presence of appropriate antibiotics, whereas AG-susceptible bacteria were grown to exponential phase in media devoid of antibiotics after which appropriate antibiotics were added and cultures were incubated for an additional 3.5 hours. The Materials and Methods section has been revised to clarify this (lines 106-112).

*“53. Figure S1c: for context, we need to know the Km conc in the media for all the groups. Based on Figure 1c, you probably have >50 ug/mL in the first group, which alone would explain the greater/faster killing since that is 10x more drug than what was manually added for group 2”*

The data in Fig. S1C complement the data depicted in Fig. 4F. For both experiments, kanamycin-resistant *A. baumannii* Tn5A7 was grown to mid-exponential phase in media supplemented with 40 μg/mL kanamycin prior to chemical killing as detailed in the Materials and Methods section. In agreement with the Reviewer, these data suggest that co-incubation with killed, kanamycin-bound *A. baumannii* may lead to an effective kanamycin concentration of >50 μg/mL. This could indeed explain why more killing of AG-naïve *A. baumannii* is observed in this group compared to any other group in which kanamycin was added in solution.

Concluding comments and suggestions:

*“1. It is unclear what the authors are proposing for AG interaction with surfactant, and the schematic (while helpful) does not adequately explain it. Do they propose that host surfactants are displacing AG from the OM? Or is the AG being released via some other mechanism, and the free AG then binds/interacts with surfactant, which then shuttles drug to kill neighboring naïve bacterial cells? Or maybe a combination of both?”*

Data included in the revised manuscript do not support the conclusion that detergents displace AGs from AG-bound bacteria (Fig. 4G). Based on this, the current working model is that AGs are released from AG-bound bacteria inside the lung by some other mechanism, and that pulmonary surfactant increases susceptibility of AG-naïve, co-infecting bacteria to free AG molecules (lines 450-455). A potential, additional role for pulmonary surfactant in the transfer of AG molecules from AG-bound bacteria to AG-naïve, co-infecting bacteria cannot be ruled out. The model in Fig. 5 has been revised accordingly.

*“2. Could you incorporate some kind of displacement assay to further prove that the OM is the reservoir? Either by adding MgSO4 or surfactant and/or detergent after AGs bind, and then demonstrate that drug can be displaced (or released) from the OM by using LC/MS to measure drug conc in supernatants, cell pellets and cell lysates both before and after displacement.”*

We thank the Reviewer for this suggestion. We have performed additional experiments to strengthen the conclusion that the Gram-negative OM is implicated as the predominant AG reservoir (Fig 3A, our response to Reviewer 1 “Specific Comment” 1, Fig. 4G, and our response to Reviewer 3 “Scientific Limitation” 1).

*“3. Could you demonstrate that the OM is the reservoir by killing the bugs before vs. after AG exposure (i.e. if the cell is still intact and there is no active process since its dead, then presumably AGs would not be internalized but would still bind to the OM if that is the reservoir)? Also, could you use OM vesicles or GMMA to isolate only that part of the cell for testing the theory?”*

The additional experiments performed raise several additional lines of evidence implicating the Gram-negative OM as the predominant AG reservoir (lines 415-428, see our response to Reviewer 3 “Concluding Comments and Suggestions” 2). We agree with the Reviewer that the proposed experiments regarding OM vesicles are interesting. However, we view them as future directions and consider them to be outside the scope of the present manuscript.

*“4. Why would AGs interact with the membrane instead of being taken up internally? How stable is the binding? Is cell permeability and/or efflux affected in R strains?”*

Please view our response to Reviewer 3 Line Comment 27 for our working hypothesis on why AGs preferentially interact with the OM. The present manuscript and one previous report indicate that the binding of AGs to the Gram-negative OM is strong enough to withstand multiple washes (Sabeti Azad *et al.*, 2020). We agree with the Reviewer that the proposed experiments would be interesting. However, we view them as future directions and consider them to be outside the scope of the present manuscript. Of note, in the AG-resistant strains used in this study, resistance is conferred by AG-modifying enzymes. In addition, AG-resistant and AG-susceptible bacteria are equally as effective at inducing AG-mediated killing of AG-naïve, co-infecting bacteria inside the murine lung (Fig. 3E). These data do not suggest that more or less AG is shuttled from the OM to the cytosol in AG-resistant strains in comparison to AG-susceptible strains. However, whether cell permeability and/or efflux is affected in general in AG-resistant strains is an interesting point for future investigation.

*“5. How does the capsule on some gram-negative bacteria come into play? Do you see the same effect in encapsulated vs. un-encapsulated strains?”*

We thank the Reviewer for this interesting suggestion. Reports indicate that bacterial capsular polysaccharides such as those produced by *P. aeruginosa* and *K. pneumoniae* are negatively charged and can bind cationic molecules such as antimicrobial peptides (AMPs) (Herasimenka *et al.*, 2005, Li *et al.*, 2013). Whether bacterial capsules contribute to AG binding and retention following *in vitro* exposure, and to what extent this might be affected by differential capsule production by the pathogens used in the present study remains to be investigated. We view these as intriguing future directions.

**REFERENCES (formatting will be finalized once final draft is agreed upon)**

Anderson MS, Robertson AD, Macher I & Raetz CR (1988) Biosynthesis of lipid A in *Escherichia coli*: identification of UDP-3-O-[(R)-3-hydroxymyristoyl]-alpha-D-glucosamine as a precursor of UDP-N2,O3-bis[(R)-3-hydroxymyristoyl]-alpha-D-glucosamine. *Biochemistry* **27**: 1908-1917.

Barb AW, Jiang L, Raetz CR & Zhou P (2007) Structure of the deacetylase LpxC bound to the antibiotic CHIR-090: Time-dependent inhibition and specificity in ligand binding. *Proc Natl Acad Sci U S A* **104**: 18433-18438.

Boselli E, Breilh D, Djabarouti S, Guillaume C, Rimmelé T, Gordien JB, Xuereb F, Saux MC & Allaouchiche B (2007) Reliability of mini-bronchoalveolar lavage for the measurement of epithelial lining fluid concentrations of tobramycin in critically ill patients. *Intensive Care Med* **33**: 1519-1523.

Dobie RA, Black FO, Pezsnecker SC & Stallings VL (2006) Hearing loss in patients with vestibulotoxic reactions to gentamicin therapy. *Arch Otolaryngol Head Neck Surg* **132**: 253-257.

Dyrda G, Boniewska-Bernacka E, Man D, Barchiewicz K & Słota R (2019) The effect of organic solvents on selected microorganisms and model liposome membrane. *Mol Biol Rep* **46**: 3225-3232.

Garneau-Tsodikova S & Labby KJ (2016) Mechanisms of Resistance to Aminoglycoside Antibiotics: Overview and Perspectives. *Medchemcomm* **7**: 11-27.

Herasimenka Y, Benincasa M, Mattiuzzo M, Cescutti P, Gennaro R & Rizzo R (2005) Interaction of antimicrobial peptides with bacterial polysaccharides from lung pathogens. *Peptides* **26**: 1127-1132.

Hood-Pishchany MI, Pham L, Wijers CD, Burns WJ, Boyd KL, Palmer LD, Skaar EP & Noto MJ (2020) Broad-spectrum suppression of bacterial pneumonia by aminoglycoside-propagated *Acinetobacter baumannii*. *PLoS Pathog* **16**: e1008374.

Isaksson B, Nilsson L, Maller R & Sörén L (1988) Postantibiotic effect of aminoglycosides on gram-negative bacteria evaluated by a new method. *J Antimicrob Chemother* **22**: 23-33.

Jackson GG, Lolans VT & Daikos GL (1990) The inductive role of ionic binding in the bactericidal and postexposure effects of aminoglycoside antibiotics with implications for dosing. *J Infect Dis* **162**: 408-413.

Kalil AC, Metersky ML, Klompas M*, et al.* (2016) Management of Adults With Hospital-acquired and Ventilator-associated Pneumonia: 2016 Clinical Practice Guidelines by the Infectious Diseases Society of America and the American Thoracic Society. *Clin Infect Dis* **63**: e61-e111.

Kwon DH & Lu CD (2006) Polyamines induce resistance to cationic peptide, aminoglycoside, and quinolone antibiotics in Pseudomonas aeruginosa PAO1. *Antimicrob Agents Chemother* **50**: 1615-1622.

Leone M, Bouadma L, Bouhemad B*, et al.* (2018) Hospital-acquired pneumonia in ICU. *Anaesth Crit Care Pain Med* **37**: 83-98.

Li Y, Weinberger DM, Thompson CM, Trzciński K & Lipsitch M (2013) Surface charge of *Streptococcus pneumoniae* predicts serotype distribution. *Infect Immun* **81**: 4519-4524.

McDonnell G & Russell AD (1999) Antiseptics and disinfectants: activity, action, and resistance. *Clin Microbiol Rev* **12**: 147-179.

Mingeot-Leclercq MP & Tulkens PM (1999) Aminoglycosides: nephrotoxicity. *Antimicrob Agents Chemother* **43**: 1003-1012.

Mouton JW, Theuretzbacher U, Craig WA, Tulkens PM, Derendorf H & Cars O (2008) Tissue concentrations: do we ever learn? *J Antimicrob Chemother* **61**: 235-237.

Naber KG, Westenfelder SR & Madsen PO (1973) Pharmacokinetics of the aminoglycoside antibiotic tobramycin in humans. *Antimicrob Agents Chemother* **3**: 469-473.

Panidis D, Markantonis SL, Boutzouka E, Karatzas S & Baltopoulos G (2005) Penetration of gentamicin into the alveolar lining fluid of critically ill patients with ventilator-associated pneumonia. *Chest* **128**: 545-552.

Radlinski LC, Rowe SE, Brzozowski R, Wilkinson AD, Huang R, Eswara P & Conlon BP (2019) Chemical Induction of Aminoglycoside Uptake Overcomes Antibiotic Tolerance and Resistance in *Staphylococcus aureus*. *Cell Chem Biol* **26**: 1355-1364.e1354.

Ramsey BW, Pepe MS, Quan JM*, et al.* (1999) Intermittent administration of inhaled tobramycin in patients with cystic fibrosis. Cystic Fibrosis Inhaled Tobramycin Study Group. *N Engl J Med* **340**: 23-30.

Sabeti Azad M, Okuda M, Cyrenne M, Bourge M, Heck MP, Yoshizawa S & Fourmy D (2020) Fluorescent Aminoglycoside Antibiotics and Methods for Accurately Monitoring Uptake by Bacteria. *ACS Infect Dis* **6**: 1008-1017.

Vidal L, Gafter-Gvili A, Borok S, Fraser A, Leibovici L & Paul M (2007) Efficacy and safety of aminoglycoside monotherapy: systematic review and meta-analysis of randomized controlled trials. *J Antimicrob Chemother* **60**: 247-257.

Wachino J & Arakawa Y (2012) Exogenously acquired 16S rRNA methyltransferases found in aminoglycoside-resistant pathogenic Gram-negative bacteria: an update. *Drug Resist Updat* **15**: 133-148.

Wei JR, Richie DL, Mostafavi M, Metzger LE, Rath CM, Sawyer WS, Takeoka KT & Dean CR (2017) LpxK Is Essential for Growth of  *Acinetobacter baumannii* ATCC 19606: Relationship to Toxic Accumulation of Lipid A Pathway Intermediates. *mSphere* **2**.

Wood MJ & Farrell W (1976) Comparison of urinary excretion of tobramycin and gentamicin in adults. *J Infect Dis* **134 Suppl**: S133-136.

Wu H, Kuzmenko A, Wan S, Schaffer L, Weiss A, Fisher JH, Kim KS & McCormack FX (2003) Surfactant proteins A and D inhibit the growth of Gram-negative bacteria by increasing membrane permeability. *J Clin Invest* **111**: 1589-1602.

Yokoyama K, Doi Y, Yamane K, Kurokawa H, Shibata N, Shibayama K, Yagi T, Kato H & Arakawa Y (2003) Acquisition of 16S rRNA methylase gene in *Pseudomonas aeruginosa*. *Lancet* **362**: 1888-1893.
